# Technology-mediated training programs for school health teams on special health care needs: a scoping review

**DOI:** 10.1590/1980-220X-REEUSP-2024-0328en

**Published:** 2025-05-09

**Authors:** Maria do Céu Coelho Monteiro Pires, Maria do Céu Aguiar Barbieri - Figueiredo, Daniela Filipa Batista Cardoso, Filipa Margarida Duque, Maria Sandra Tricas-Sauras, Mirko Prosen, Eva Guilherme Menino

**Affiliations:** 1 Universidade do Porto, Instituto de Ciências Biomédicas Abel Salazar, Porto, Portugal.; 2 Escola Superior de Saúde da Cruz Vermelha Portuguesa - Lisboa, Lisboa, Portugal.; 3 Universidad de Huelva, Facultad Enfermería, Huelva, Espanha.; 4 Centro Português para a Prática Baseada na Evidência, Unidade de Investigação em Ciências da Saúde: Enfermagem, Escola Superior de Enfermagem de Coimbra, Coimbra, Portugal.; 5 Escola Superior de Enfermagem de Coimbra, Unidade de Investigação em Ciências da Saúde: Enfermagem, Coimbra, Portugal.; 6 Université Libre de Bruxelles, École de Santé Publique, Centre de Recherche sur les Approches Sociales de la Santé, Bruxelles, Belgique.; 7 University of Primorska, Faculty of Health Sciences, Department of Nursing, Izola, Slovenia.; 8 Center for Innovative Care and Health Technology, Escola Superior de Saúde, Instituto Politécnico de Leiria, Leiria, Portugal

**Keywords:** School Health Services, Child Health, Professional Training, Educational Technology, School Nursing, Serviços de Saúde Escolar, Saúde da Criança, Capacitação Profissional, Tecnologia Educacional, Serviços de Enfermagem Escolar

## Abstract

**Objective:**

To map technology-mediated training programs for school health teams that address special health needs in the school environment and to identify their characteristics.

**Method:**

The review followed the JBI methodology. Eight databases were searched for published and gray literature. Studies published in Portuguese, English or Spanish since 2000 were included to capture emerging training programs.

**Results:**

Of the 1,106 studies identified, 29 were reviewed in full and eight were included in the final analysis. All studies were carried out in the United States of America. Program topics included chronic health conditions such as diabetes, asthma, procedures, as well as emergency situations, all aimed at school nurses and based on a conceptual or pedagogical framework. The programs included thematic modules of various lengths and offered online and hybrid training through various digital educational resources.

**Conclusions:**

Programs focused on chronic health conditions and assessed professionals' knowledge, skill development, self-efficacy, and confidence; few studies provided a detailed exploration of the underlying pedagogical models and did not use formative assessment.

## RESUMO

**Objetivo:**

Mapear programas de treinamento mediados por tecnologia para equipes de saúde escolar que abordem necessidades especiais de saúde no ambiente escolar e identificar suas características.

**Método:**

A revisão seguiu a metodologia do JBI. Foram pesquisadas oito bases de dados para literatura publicada e cinzenta. Estudos publicados em português, inglês ou espanhol desde 2000 foram incluídos para capturar programas de treinamento emergentes.

**Resultados:**

Dos 1.106 estudos identificados, 29 foram revisados na íntegra e oito foram incluídos na análise final. Todos os estudos foram realizados nos Estados Unidos da América. Os tópicos do programa incluíam condições crônicas de saúde, como diabetes, asma, procedimentos e situações de emergência, todos voltados para enfermeiros escolares e baseados em uma estrutura conceitual ou pedagógica. Os programas incluíam módulos temáticos de durações variadas e ofereciam treinamento on-line e híbrido por meio de vários recursos educacionais digitais.

**Conclusões:**

Os programas concentraram-se em condições crônicas de saúde e avaliaram o conhecimento, o desenvolvimento de habilidades, a autoeficácia e a confiança dos profissionais; poucos estudos forneceram uma exploração detalhada dos modelos pedagógicos subjacentes e não usaram avaliação formativa.

## INTRODUCTION

School plays a central role in children and young people’s lives. In addition to promoting education, it is also responsible for promoting health and well-being, as students spend a large part of their time in the school environment^(1–4)^. The American Academy of Pediatrics (AAP) defines children and youth with special health care needs (CSHCN) as those who have, or are at risk of developing chronic conditions that require health care and specialized services^(5)^. These children face disadvantages in the school environment, with a higher risk of exclusion from educational and social activities, which can negatively impact their academic performance^(6–9)^. The integration of health and education is therefore essential to create a universal health cycle, ensuring access to inclusive and quality education for all children, regardless of their health conditions^(10,11)^.

According to the World Health Organization (WHO), school health services are provided by health professionals within schools or in collaboration with external services^(4)^. Although the composition of school health teams is not widely detailed in the literature, it typically includes nurses, doctors, and psychologists^(3,4,12)^. In particular, school nurses, who require specialized training and continuous professional development, play an essential role in daily support for students’ health needs^(2,3,13)^.

Digital transformation has been a crucial ally in this process, facilitating access to health information and training, and exploring new tools that make education more inclusive and adapted to the digital age^(14,15)^. The COVID-19 pandemic further reinforced this importance, accelerating the incorporation of technologies to promote learning^(15,16)^.

Most interventions considered essential in school health services concern health promotion and health education, compared to those related to the inclusion of CSHCN^(17)^. In addition, one of the most frequently reported challenges for school health services is the lack of training and continuing professional development opportunities^(4)^.

Training programs are structured sets of interrelated activities designed to develop knowledge, attitudes, and skills in specific areas^(18)^. These programs include educational interventions, in this case mediated by technologies, that foster learning and capacity building, enhancing participants’ performance and competencies.

In this study, the term technology refers to educational technology as the application of technological tools and resources to improve teaching and learning. It encompasses the use of various digital tools, software and devices to improve educational outcomes and experiences^(19)^.

The literature is scattered in relation to the pedagogical process and the conceptual framework of training, specifically regarding technology-mediated training. The main characteristics of these programs and the link between learning outcomes and the assessment tools used are also not well characterized.

This scoping review aimed to map technology-mediated training programs for school health teams, focusing on special health care needs in the school setting. The specific review questions were: What pedagogical models, theories, or conceptual principles have been used to design these training programs? Do these training programs address specific health conditions? What is the organization of the training process (learning objectives, program, timetable, modality, type of technology, and participants) and the teaching and learning strategies? What learning outcomes were assessed, and what instruments were used to assess the desired learning objectives?

The specific reason for choosing a scoping review is because it aims to map the existing evidence underlying a research area, identify gaps in the existing evidence, and build a precursor exercise that justifies and informs the conduct of a systematic literature review^(20)^.

## METHOD

A search conducted on September 13, 2021, in MEDLINE (PubMed) and the JBI Evidence Synthesis, revealed that no literature review is available or being developed on technology-mediated training programs for school health teams focused on special health needs. Thus, opportunities for technology-mediated training for school health teams should be identified for them to develop skills in their practice and find gaps for future research in the field of school nursing.

### Design of Study

We followed the JBI methodology for scoping reviews, and the recommendations of the Preferred Reporting Items for Systematic reviews and Meta-Analyses extension for Scoping Reviews (PRISMA-ScR) Checklist^(21,22)^ associated with the PRISMA 2020 Flowchart^(23)^. The quality of the studies was not particularly assessed as this is a scoping review. The review protocol was carried out and registered in the Open Science Framework (OSF) with the review title and DOI 10.17605/OSF.IO/53Z89.

### Data Sources and Research Strategy

The review question was formulated according to the PCC framework, with Population (P) being school health teams, Concept (C) focusing on technology-mediated training programs addressing special health care needs, and Context (C) encompassing any location where training can take place. The types of evidence sources included primary studies utilizing qualitative, quantitative, or mixed methods, as well as systematic reviews. The review was restricted to studies published in Portuguese, English, or Spanish from 2000.

The review included studies involving healthcare professionals from school health teams, particularly those qualified in health promotion and supporting CSHCN. It also included technology-mediated training programs aimed at enhancing the skills of school health teams in managing CSHCN and chronic health conditions, namely educational interventions, such as sessions, seminars, lectures, and continuous professional development, specifically designed for healthcare professionals in the school context. The context included any training environment, and the selected chronological period reflects the technological advancements of the early 21st century, highlighting the evolution of technology in education and training. Studies targeting non-healthcare professionals, such as teachers, and studies lacking clear identification of training programs were excluded.

A three-step search strategy was used. First, an initial search on MEDLINE (via PubMed), CINAHL (via EBSCO) using general keywords, such as “School Health” AND technolog* AND (lifelong or Continuing) were conducted. The results of this initial search were analyzed to identify other keywords and index terms to be used in final search strategy. These terms were organized in a logic grid that was the basis to conduct the second phase of the search. In the second phase, five databases were searched: MEDLINE (via PubMed), CINAHL (via EBSCO), SCOPUS, Psychology and Behavioral Sciences Collection (via EBSCO) and LILACS, applying the keywords and indexed terms identified previously and adapted for each database included.

The search of grey literature was conducted in the *Repositório Científico de Acesso Aberto de Portugal* [Open Access Scientific Repository of Portugal] (RCAAP), *Catálogo de Teses & Dissertações* [Theses and Dissertation Catalog] (CAPES), and Mednar. Additionally, three official and governmental websites dealing with the topic, specifically the Centers for Disease Control and Prevention (CDC) - Health School, the National Association of School Nurses (NASN), and the New Zealand School Nurses (NZ+SN) were searched. In the third phase, the reference list of the selected studies for additional studies was searched, as well as the gray literature. The full search strategy for each database and sources of grey literature is detailed in Chart 1.

**Chart 1:** Scoping review search strategies – Coimbra, Portugal, 2021–2022.

MEDLINE via PubMed: Searched on November 11, 2021		
ID	Search strategy	
#19	Search: (((“school nurses”[Title/Abstract] OR “School Health”[Title/Abstract] OR “School-Based Health”[Title/Abstract] OR “School Based Services”[Title/Abstract] OR “School Based Service”[Title/Abstract] OR “School-Based Service”[Title/Abstract] OR “School-Based Services”[Title/Abstract] OR “School Based Health”[Title/Abstract] OR “school nurse”[Title/Abstract]) OR (“School Health Services”[Mesh])) AND ((Continuing[Title/Abstract] OR “lifelong learning”[Title/Abstract] OR “professional development”[Title/Abstract]) OR (“Education, Continuing”[Mesh]))) AND (((((((Technolog*[Title/Abstract] OR Computer*[Title/Abstract] OR Online[Title/Abstract] OR Smartphone*[Title/Abstract] OR Electronic*[Title/Abstract] OR Virtual[Title/Abstract] OR Augmented[Title/Abstract] OR Mobile[Title/Abstract] OR Gamification[Title/Abstract] OR Platform*[Title/Abstract] OR Internet[Title/Abstract] OR e-learn*[Title/Abstract] OR MOOC[Title/Abstract] OR Distance[Title/Abstract] OR Hybrid[Title/Abstract] OR Digital[Title/Abstract] OR Blend*[Title/Abstract]) OR (“Education, Distance”[Mesh])) OR (“Computer User Training”[Mesh])) OR (“Computer Simulation”[Mesh])) OR (“Computer-Assisted Instruction”[Mesh])) OR (“Educational Technology”[Mesh])) OR (“Digital Technology”[Mesh])) Filters: English, Portuguese, Spanish	79
#18	Search: (((“school nurses”[Title/Abstract] OR “School Health”[Title/Abstract] OR “School-Based Health”[Title/Abstract] OR “School Based Services”[Title/Abstract] OR “School Based Service”[Title/Abstract] OR “School-Based Service”[Title/Abstract] OR “School-Based Services”[Title/Abstract] OR “School Based Health”[Title/Abstract] OR “school nurse”[Title/Abstract]) OR (“School Health Services”[Mesh])) AND ((Continuing[Title/Abstract] OR “lifelong learning”[Title/Abstract] OR “professional development”[Title/Abstract]) OR (“Education, Continuing”[Mesh]))) AND (((((((Technolog*[Title/Abstract] OR Computer*[Title/Abstract] OR Online[Title/Abstract] OR Smartphone*[Title/Abstract] OR Electronic*[Title/Abstract] OR Virtual[Title/Abstract] OR Augmented[Title/Abstract] OR Mobile[Title/Abstract] OR Gamification[Title/Abstract] OR Platform*[Title/Abstract] OR Internet[Title/Abstract] OR e-learn*[Title/Abstract] OR MOOC[Title/Abstract] OR Distance[Title/Abstract] OR Hybrid[Title/Abstract] OR Digital[Title/Abstract] OR Blend*[Title/Abstract]) OR (“Education, Distance”[Mesh])) OR (“Computer User Training”[Mesh])) OR (“Computer Simulation”[Mesh])) OR (“Computer-Assisted Instruction”[Mesh])) OR (“Educational Technology”[Mesh])) OR (“Digital Technology”[Mesh])) Filters: English, Portuguese	78
#17	Search: (((“school nurses”[Title/Abstract] OR “School Health”[Title/Abstract] OR “School-Based Health”[Title/Abstract] OR “School Based Services”[Title/Abstract] OR “School Based Service”[Title/Abstract] OR “School-Based Service”[Title/Abstract] OR “School-Based Services”[Title/Abstract] OR “School Based Health”[Title/Abstract] OR “school nurse”[Title/Abstract]) OR (“School Health Services”[Mesh])) AND ((Continuing[Title/Abstract] OR “lifelong learning”[Title/Abstract] OR “professional development”[Title/Abstract]) OR (“Education, Continuing”[Mesh]))) AND (((((((Technolog*[Title/Abstract] OR Computer*[Title/Abstract] OR Online[Title/Abstract] OR Smartphone*[Title/Abstract] OR Electronic*[Title/Abstract] OR Virtual[Title/Abstract] OR Augmented[Title/Abstract] OR Mobile[Title/Abstract] OR Gamification[Title/Abstract] OR Platform*[Title/Abstract] OR Internet[Title/Abstract] OR e-learn*[Title/Abstract] OR MOOC[Title/Abstract] OR Distance[Title/Abstract] OR Hybrid[Title/Abstract] OR Digital[Title/Abstract] OR Blend*[Title/Abstract]) OR (“Education, Distance”[Mesh])) OR (“Computer User Training”[Mesh])) OR (“Computer Simulation”[Mesh])) OR (“Computer-Assisted Instruction”[Mesh])) OR (“Educational Technology”[Mesh])) OR (“Digital Technology”[Mesh])) Filters: English	78
#16	Search: (((“school nurses”[Title/Abstract] OR “School Health”[Title/Abstract] OR “School-Based Health”[Title/Abstract] OR “School Based Services”[Title/Abstract] OR “School Based Service”[Title/Abstract] OR “School-Based Service”[Title/Abstract] OR “School-Based Services”[Title/Abstract] OR “School Based Health”[Title/Abstract] OR “school nurse”[Title/Abstract]) OR (“School Health Services”[Mesh])) AND ((Continuing[Title/Abstract] OR “lifelong learning”[Title/Abstract] OR “professional development”[Title/Abstract]) OR (“Education, Continuing”[Mesh]))) AND (((((((Technolog*[Title/Abstract] OR Computer*[Title/Abstract] OR Online[Title/Abstract] OR Smartphone*[Title/Abstract] OR Electronic*[Title/Abstract] OR Virtual[Title/Abstract] OR Augmented[Title/Abstract] OR Mobile[Title/Abstract] OR Gamification[Title/Abstract] OR Platform*[Title/Abstract] OR Internet[Title/Abstract] OR e-learn*[Title/Abstract] OR MOOC[Title/Abstract] OR Distance[Title/Abstract] OR Hybrid[Title/Abstract] OR Digital[Title/Abstract] OR Blend*[Title/Abstract]) OR (“Education, Distance”[Mesh])) OR (“Computer User Training”[Mesh])) OR (“Computer Simulation”[Mesh])) OR (“Computer-Assisted Instruction”[Mesh])) OR (“Educational Technology”[Mesh])) OR (“Digital Technology”[Mesh])) Filters: Spanish	1
#15	Search: (((“school nurses”[Title/Abstract] OR “School Health”[Title/Abstract] OR “School-Based Health”[Title/Abstract] OR “School Based Services”[Title/Abstract] OR “School Based Service”[Title/Abstract] OR “School-Based Service”[Title/Abstract] OR “School-Based Services”[Title/Abstract] OR “School Based Health”[Title/Abstract] OR “school nurse”[Title/Abstract]) OR (“School Health Services”[Mesh])) AND ((Continuing[Title/Abstract] OR “lifelong learning”[Title/Abstract] OR “professional development”[Title/Abstract]) OR (“Education, Continuing”[Mesh]))) AND (((((((Technolog*[Title/Abstract] OR Computer*[Title/Abstract] OR Online[Title/Abstract] OR Smartphone*[Title/Abstract] OR Electronic*[Title/Abstract] OR Virtual[Title/Abstract] OR Augmented[Title/Abstract] OR Mobile[Title/Abstract] OR Gamification[Title/Abstract] OR Platform*[Title/Abstract] OR Internet[Title/Abstract] OR e-learn*[Title/Abstract] OR MOOC[Title/Abstract] OR Distance[Title/Abstract] OR Hybrid[Title/Abstract] OR Digital[Title/Abstract] OR Blend*[Title/Abstract]) OR (“Education, Distance”[Mesh])) OR (“Computer User Training”[Mesh])) OR (“Computer Simulation”[Mesh])) OR (“Computer-Assisted Instruction”[Mesh])) OR (“Educational Technology”[Mesh])) OR (“Digital Technology”[Mesh]))	79
#14	Search: ((((((Technolog*[Title/Abstract] OR Computer*[Title/Abstract] OR Online[Title/Abstract] OR Smartphone*[Title/Abstract] OR Electronic*[Title/Abstract] OR Virtual[Title/Abstract] OR Augmented[Title/Abstract] OR Mobile[Title/Abstract] OR Gamification[Title/Abstract] OR Platform*[Title/Abstract] OR Internet[Title/Abstract] OR e-learn*[Title/Abstract] OR MOOC[Title/Abstract] OR Distance[Title/Abstract] OR Hybrid[Title/Abstract] OR Digital[Title/Abstract] OR Blend*[Title/Abstract]) OR (“Education, Distance”[Mesh])) OR (“Computer User Training”[Mesh])) OR (“Computer Simulation”[Mesh])) OR (“Computer-Assisted Instruction”[Mesh])) OR (“Educational Technology”[Mesh])) OR (“Digital Technology”[Mesh])	2,493,775
#13	Search: “Digital Technology”[Mesh] Sort by: Most Recent	349
#12	Search: “Educational Technology”[Mesh] Sort by: Most Recent	112,531
#11	Search: “Computer-Assisted Instruction”[Mesh] Sort by: Most Recent	12,258
#10	Search: “Computer Simulation”[Mesh] Sort by: Most Recent	265,374
#9	Search: “Computer User Training”[Mesh] Sort by: Most Recent	2,041
#8	Search: “Education, Distance”[Mesh] Sort by: Most Recent	5,550
#7	Search: Technolog*[Title/Abstract] OR Computer*[Title/Abstract] OR Online[Title/Abstract] OR Smartphone*[Title/Abstract] OR Electronic*[Title/Abstract] OR Virtual[Title/Abstract] OR Augmented[Title/Abstract] OR Mobile[Title/Abstract] OR Gamification[Title/Abstract] OR Platform*[Title/Abstract] OR Internet[Title/Abstract] OR e-learn*[Title/Abstract] OR MOOC[Title/Abstract] OR Distance[Title/Abstract] OR Hybrid[Title/Abstract] OR Digital[Title/Abstract] OR Blend*[Title/Abstract]	2,205,126
#6	Search: (Continuing [Title/Abstract] OR “lifelong learning”[Title/Abstract] OR “professional development”[Title/Abstract]) OR (“Education, Continuing”[Mesh])	139,978
#5	Search: (“school nurses”[Title/Abstract] OR “School Health”[Title/Abstract] OR “School-Based Health”[Title/Abstract] OR “School Based Services”[Title/Abstract] OR “School Based Service”[Title/Abstract] OR “School-Based Service”[Title/Abstract] OR “School-Based Services”[Title/Abstract] OR “School Based Health”[Title/Abstract] OR “school nurse”[Title/Abstract]) OR (“School Health Services”[Mesh])	28,076
#4	Search: “Education, Continuing”[Mesh] Sort by: Most Recent	62,175
#3	Search: Continuing [Title/Abstract] OR “lifelong learning”[Title/Abstract] OR “professional development”[Title/Abstract]	91,552
#2	Search: “School Health Services”[Mesh] Sort by: Most Recent	23,930
#1	Search: “school nurses”[Title/Abstract] OR “School Health”[Title/Abstract] OR “School-Based Health”[Title/Abstract] OR “School Based Services”[Title/Abstract] OR “School Based Service”[Title/Abstract] OR “School-Based Service”[Title/Abstract] OR “School-Based Services”[Title/Abstract] OR “School Based Health”[Title/Abstract] OR “school nurse”[Title/Abstract]	11,013
**SCOPUS:** Searched on November 11, 2021		
Search strategy		
( TITLE-ABS-KEY ( “school nurses” OR “School Health” OR “School-Based Health” OR “School Based Services” OR “School Based Service” OR “School-Based Service” OR “School-Based Services” OR “School Based Health” OR “school nurse” ) AND TITLE-ABS-KEY ( continuing OR “lifelong learning” OR “professional development” ) AND TITLE-ABS-KEY ( technolog* OR computer* OR online OR smartphone* OR electronic* OR virtual OR augmented OR mobile OR gamification OR platform* OR internet OR e-learn* OR mooc OR distance OR hybrid OR digital OR blend* ) )		118
**Psychology & Behavioral Sciences Collection via EBSCOhost:** Searched on November 11, 2021		
Search strategy		
S1 AND S2 AND S3		4
TI ( Technolog* OR Computer* OR Online OR Smartphone* OR Electronic* OR Virtual OR Augmented OR Mobile OR Gamification OR Platform* OR Internet OR e-learn* OR MOOC OR Distance OR Hybrid OR Digital OR Blend* ) OR AB ( Technolog* OR Computer* OR Online OR Smartphone* OR Electronic* OR Virtual OR Augmented OR Mobile OR Gamification OR Platform* OR Internet OR e-learn* OR MOOC OR Distance OR Hybrid OR Digital OR Blend* )		93,277
TI (Continuing OR “lifelong learning” OR “professional development”) OR AB (Continuing OR “lifelong learning” OR “professional development”)		8,284
TI ( “school nurses” OR “School Health” OR “School-Based Health” OR “School Based Services” OR “School Based Service” OR “School-Based Service” OR “School-Based Services” OR “School Based Health” OR “school nurse” ) OR AB ( “school nurses” OR “School Health” OR “School-Based Health” OR “School Based Services” OR “School Based Service” OR “School-Based Service” OR “School-Based Services” OR “School Based Health” OR “school nurse” )		1,386
**CHINAL** Complete via EBSCOhost**:** Searched on November 6, 2021		
Search strategy		
S9 AND S18		72
S10 OR S11 OR S12 OR S13 OR S14 OR S15 OR S16 OR S17		529,504
(MH “Digital Technology”)		520
(MH “Educational Technology”)		2,270
(MH “Programmed Instruction+”)		8,693
(MH “Gamification”)		108
(MH “Computer Simulation+”)		24,615
(MH “Computer User Training”)		790
(MH “Education, Non-Traditional+”)		12,192
TI ( Technolog* OR Computer* OR Online OR Smartphone* OR Electronic* OR Virtual OR Augmented OR Mobile OR Gamification OR Platform* OR Internet OR e-learn* OR MOOC OR Distance OR Hybrid OR Digital OR Blend* ) OR AB ( Technolog* OR Computer* OR Online OR Smartphone* OR Electronic* OR Virtual OR Augmented OR Mobile OR Gamification OR Platform* OR Internet OR e-learn* OR MOOC OR Distance OR Hybrid OR Digital OR Blend* )		506,762
S5 AND S8		572
S6 OR S7		77,866
(MH “Education, Continuing+”)		36,102
TI (Continuing OR “lifelong learning” OR “professional development”) OR AB (Continuing OR “lifelong learning” OR “professional development”)		50,779
S1 OR S2 OR S3 OR S4		28,980
(MH “School Health Services+”)		24,084
(MH “School Health”)		4,067
(MH “National Association of School Nurses”)		557
TI ( “school nurses” OR “School Health” OR “School-Based Health” OR “School Based Services” OR “School Based Service” OR “School-Based Service” OR “School-Based Services” OR “School Based Health” OR “school nurse” ) OR AB ( “school nurses” OR “School Health” OR “School-Based Health” OR “School Based Services” OR “School Based Service” OR “School-Based Service” OR “School-Based Services” OR “School Based Health” OR “school nurse” )		7,426
**LILACS:** Searched on March 21, 2022		
ID	Search strategy	
	(“school health”) AND (continuing)	25
**RCAAP:** Searched on March 21, 2022		
ID	Search strategy	
	“school health”	52
**CAPES:** Searched on March 21, 2022		
ID	Search strategy	
	“school health” AND continuing	23
**Mednar:** Searched on March 29, 2022		
ID	Search strategy	
	“school health” AND continuing AND technology	733

Duplicates were eliminated using the software Mendeley Reference Manager. The software Rayyan Qatar Computing Research Institute (Rayyan QCRI) was used to assist in archiving, organizing, and selecting the articles for title and abstract.

### Study Selection and Data Extraction

The study selection process was conducted independently in two stages by two reviewers, with a third reviewer resolving conflicts. The first stage involved reviewing titles and abstracts to exclude articles that did not meet the inclusion criteria. If the eligibility of a study was unclear, it was still advanced to the second stage for further evaluation. In this second stage, all eligible studies were retrieved in full, thoroughly read, and analyzed. Data were independently extracted by two reviewers. In case of disagreement, a third reviewer intervened. Extracted data included details about the authors, year, country, study type, and sample characteristics, data on training programs, including covering participants (number and type), conceptual and pedagogical framework, objectives, curriculum, schedule, modality, technologies used, teaching strategies, and assessment. Data were organized in a table developed by the authors. The data extraction form was pre-tested, and inconsistencies were discussed with senior researchers to reach a consensus.

### Ethical Aspects

No ethical review was required since the study used publicly available secondary data, and all copyright regulations for the cited research were observed.

## RESULTS

**Figure 1:**
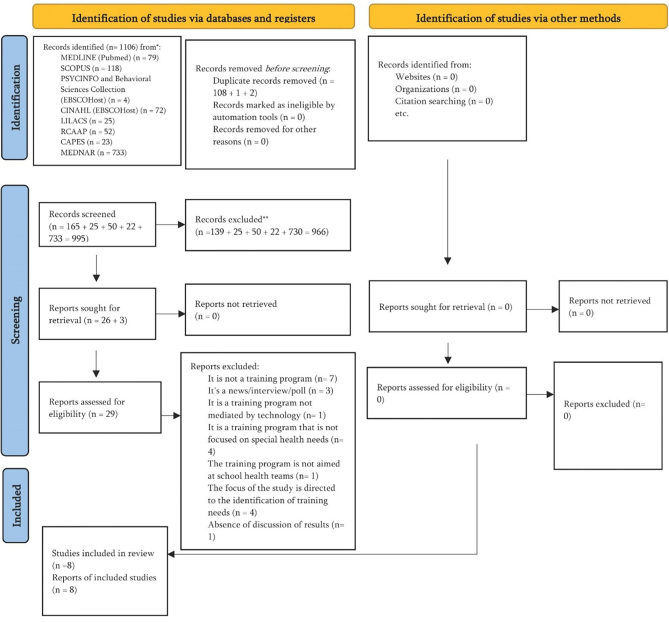
PRISMA diagram of the article search and selection stages^(23)^.

From a total of 1,106 potentially relevant articles initially identified, 111 duplicates, as well as 966 articles in the title and abstract screening, and 21 in the full-text review were excluded. Eight articles met the established inclusion criteria and were selected for data extraction. Figure 1 illustrates the study selection process, with documented reasons for exclusions.

The findings of the review were synthesized narratively. The main characteristics of the studies are summarized in Chart 2, followed by an analysis that describes the relationship between these characteristics and the objectives and questions of the review. The categories and characteristics analyzed include: the characterization of the studies and the conceptual framework of the training programs; the organization of the training process (objectives, program, schedule, modality, and technology used); the teaching and learning strategies; and the evaluation methods.

**Chart 2:** Detailed descriptive characteristics of the included studies (n = 8) – Lisboa, Portugal, 2023.

Citation, country	Design	Objective	Population sample	Guides/Framework	Training programs characteristics	Teaching and learning strategies	Assessment practices
1 Jean A. Bachman and Kuei-Hsiang Hsueh, 2008, United States^(24)^.	Program evaluation post-test intervention.	Develop and evaluate an online program for school nurses on diabetes care management for children in school.	N = 19 school nurses.	“Diabetes Management in the School Setting”^(25)^. NIH/CDC’s “Helping the Student with Diabetes Succeed”^(26)^. Developed based on Rogers’ diffusion of innovations theory^(27)^.	3 lessons over 2-3 weeks on Blackboard® covering childhood diabetes, school management, insulin pumps, and the school nurse’s role.	Engaged with online PowerPoint® presentations. Participated in three discussion boards. Posted written responses. Moderated by faculty.	Includes formative evaluation, learning objectives assessment, and feedback on content, teaching, and training format preferences.
2 Elgie et al., 2010, United States^(28)^.	Experimental after-only posttest design.	To evaluate the effectiveness of a computer-assisted emergency preparedness course for school nurses.	N = 42 school nurses.	Situated-cognitive learning theory^(29)^.	Online course on computer-assisted instruction; evaluates scene safety and stabilization skills.	15 online emergency preparedness modules focusing on decision-making with immediate feedback and skills demonstrations.	Knowledge and skills were assessed through an exam, confidence survey, and OMES, with videos evaluated by physicians unaware of the group.
3 Heidi Putman-Casdorph and Susan Pinto 2011, United States^(30)^.	A quasi-experimental design with pre-and post-test intervention evaluation.	Develop and evaluate distance learning for continuing asthma education for school nurses in West Virginia.	N= 20 school nurses.	Basic asthma information following the National Institutes of Health National Asthma Education Prevention program. Asthma Care Guidelines^(31)^.	Pilot Asthma Education Module: Delivered via web conferencing technology (Wimba Live Classroom). Pathophysiology of asthma, medications, and treatment devices.	Group 1: Received a recorded asthma education module. Group 2: Received the module live with an online instructor. Nurses earned continuing education units upon completion.	Asthma knowledge and confidence were assessed using the SHPQ, with intervention groups completing it pre- and post-module, and the control group separately.
4 Cicutto et al., 2016, United States^(32)^.	A modified Delphi and Program evaluation with pre-and post-test intervention.	To develop and implement a competency-based framework for a continuing education curriculum for school nurses.	N=40 school nurses.	The National Asthma Educator Certification Board’s Certified Asthma Educator competencies^(33)^ and the Healthy Learner Model(34,35).	The hybrid model combined an online asthma program with a local workshop, featuring 12 self-paced units on the Asthma and Allergy Foundation’s website. Trainees completed training at their own pace.	School nurses completed the online Asthma Management course and attended local workshops on asthma care, featuring case studies and hands-on inhaler assessments.	Knowledge, self-confidence, and inhaler skills were assessed, with self-efficacy rated from 0 to 4. The online course included a quiz and evaluated satisfaction.
5 Lisa Blackmon-Jones, 2016, United States^(36)^.	Program evaluation with pre-and post-test intervention.	To share the findings of implementing a standardized, blended approach to school nurse orientation.	N=20 school nurses.	According to Patricia Benner’s theoretical framework^(37)^.	The blended orientation covered Diabetes, Asthma, Anaphylaxis, Catheterizations, Tube feedings, Seizures, Ventilators, Tracheostomy care, and mental health.	The program features strategies like traditional classes and skills labs, with state-mandated screening certifications completed at the start of the year for effective follow-up.	Participants noted improvements in training and mentorship. Data collection included self-assessments, and a nurse specialist assessed proficiency after the orientation.
6 Rhodes et al., 2019, United States^(38)^.	Program evaluation with pre-and post-test intervention.	To assess the effectiveness of a rapid e-learning module for school nurse professional development in school-based diabetes management.	N = 1127 School nurses.	The latest school-based diabetes care guidelines from the National Diabetes Education Program were designed using the ICARE framework for e-learning in nursing education^(39)^.	The rapid e-learning module addressed diabetes management in schools, including updates on equipment, dietary practices, psychological impacts, type 2 management, and three staff training levels.	The email included a link, cover letter, rapid e-learning module with a pre-post test, and a certificate. A reminder encouraged summer completion, and the Extend phase provided links to credible medical resources.	Knowledge of diabetes management was assessed with multiple-choice and true-false questions from the e-learning module, which included “how-to” sections and understanding checks.
7 Shimasaki et al., 2019, United States^(40)^.	Program evaluation with pre-and post-test intervention.	Evaluate the impact of the “School Nurses Managing Diabetes Care” series on Colorado school nurses’ collaboration and self-efficacy.	N = 60 School nurses.	The student-centered Framework for 21st Century School Nursing Practice™ highlights student-focused nursing, supported by key professional principles^(41)^ and intensive diabetes management guidelines^(42)^.	The program featured telehealth sessions on diabetes care, with a 4-week ECHO series of two cohorts, each having four weekly 1-hour sessions.	Sessions support peer learning through case presentations, brief didactic talks, and are moderated by a facilitator, allowing live or chat questions.	Sessions promote peer learning, with self-efficacy and satisfaction ratings, open-ended feedback on practice changes and barriers, and an online quiz via Qualtrics.
8 American Diabetes Association (ADA) 2021, United States^(43)^.	Training Website.	Prepare and educate school staff to provide needed care to students with diabetes.		The updated guide online helping the student with diabetes succeed: a guide for school personnel.	The website offers self-paced resources for school staff on diabetes care, including guidance, workshops, and tools.	The ADA School Diabetes Care Tasks is a 19-module training program for school nurses to train non-clinical staff in diabetes care.	A post-test is available at the end of each module from the program School Diabetes Care Tasks: What Key Personnel Need to Know.

The studies reviewed span from 2008 to 2021, with most conducted post-2016 (n = 5) and all in the United States of America (USA). Most applied a quantitative approach (n = 6) for evaluating the effectiveness of the training programs^(24,28,30,36,38,40)^ while one used a mixed-methods approach^(32)^. Being a program from the American Diabetes Association website, the article does not specify the study type^(43)^.

Seven training programs addressed chronic health conditions. Four focused on diabetes^(24,38,40,43)^, two on asthma^(30,32)^ , one covered various chronic conditions, and one, emergency preparedness^(28,36)^. All studies were centered on school health nurses and adhered to theoretical-methodological frameworks, including clinical practice guides^(24,30,32,38,40,43)^, theoretical references from nursing and other disciplines^(24,28,32,36,40)^, and innovative educational models using technology^(24,38)^.

The overall objectives of collected studies focused mostly on evaluating effectiveness and developing technology-supported training^(24,28,30,32,38,40)^. Specific objectives targeted diabetes management^(24,38,40)^, asthma management^(32)^ and other chronic conditions^(36)^, while some studies lacked explicit objectives^(28,30,43)^.

The program structure included topics such as diabetes control^(24,38,40,43)^, emergency procedures^(28)^, asthma control ^(30,32)^, and procedures for chronic conditions^(36)^. Programs were categorized into e-learning and blended learning formats, with most of them being delivered asynchronously^(24,30,38,43)^, with synchronous sessions offered via videoconferencing^(28,30,40)^. All of them were organized into thematic modules with varying durations. Predominantly, short training activities were identified, such as one day of in-service training, sessions lasting two to ten hours, and four weekly sessions of one hour each^(28,30,32,40)^. Learning took place over an eight-month period within a single program^(36)^. One study did not specify the time required to complete the activities but recommended completion during the summer^(38)^. The ADA website’s content was accessible unlimited number of times, allowing learners to set their own learning pace.

Educational strategies relied on technology, such as learning management systems^(24,30,32)^, personalized email resources^(38)^, and collaborative online tools^(24)^. Case-based learning was emphasized through both asynchronous and synchronous sessions^(40)^. Audiovisual scenarios and immediate feedback were utilized to enhance learning experiences^(28)^. ADA’s website offered a range of resources, including virtual workshops, case studies, and competency checklists for school nurses^(43)^.

Blended learning formats combined online modules with face-to-face problem-based workshops^(32)^. Innovative strategies, such as skills labs and computer-based documentation training were also used^(36)^. Two studies controlled the number of participants to ensure quality^(24,40)^.

The evaluation focused on self-efficacy, confidence, knowledge acquisition, and skills development^(24,28,30,32,36,38,40)^. Program quality was assessed in three studies^(24,36,40)^, with summative assessments often applied. One study highlighted formative assessments^(24)^. Various evaluation tools were employed, such as Likert scales, true-false questions, and visual analog scales. Summative assessments included filmed simulated scenarios and case management ^(28,36)^.

## DISCUSSION

This study showed essential elements of technology-mediated training programs for school health professionals. The objectives and research questions are highlighted here. Although our research was global and covered three languages, all studies identified were conducted in the USA and focused on school nurses. This is likely related to the distinct role these professionals play in the American context. In the USA, school nurses are essential for promoting student health, well-being, and educational success, as they are often the only healthcare providers within schools^(3,13,44,45)^. The NASN, established in 1968, reinforces this structured approach by providing guidelines such as the 21^st^ Century School Nursing Practice™ model and advocating for the full-time presence of school nurses^(41,46)^. With approximately 132,300 school nurses in the USA^(47)^, ongoing investment in their professional development is supported by national assessment systems, such as the National Survey of Children’s Health^(48)^, which inform school health policies. In contrast, Europe shows a significant gap in research on this topic, despite the recommendations from the European Network of Health Promoting Schools, which emphasizes the urgent need for further investigation and the implementation of health integration policies in schools^(49)^. Furthermore, in other regions of the world, such as Latin America, Africa, and parts of Asia, structured training programs in this field of school health have also not been identified.

This scoping review included studies published in Portuguese, English, and Spanish, intending to capture a comprehensive view of global practices. However, despite this inclusive approach, all included studies originated from the United States, which demonstrates a significant geographical bias in the literature. This limitation reflects the overrepresentation of English-speaking contexts and highlights the lack of studies from regions such as Latin America, which share linguistic similarities but are underrepresented in the global research corpus.

Latin America presents a unique opportunity for research due to its high ethnic and cultural diversity and distinct social determinants of health^(50)^. Research from this region has been sparse, not only in the domain of school health training but also across other public health domains^(51)^. Addressing these gaps requires a conscious effort to include diverse voices and research outputs, thereby promoting a more comprehensive understanding of global health challenges and solutions. Expanding the geographic scope of research is crucial, not only to ensure inclusivity but also to tailor interventions that are culturally and contextually relevant.

Data revealed a significant gap in the pedagogical structures of technology-mediated training programs. Although programs integrate technology for remote learning, most do not align with established pedagogical models, often converting traditional practices into virtual formats^(52)^. Only two programs incorporate pedagogical models: the ICARE model - Introduction, Connect, Apply, Reflect, and Extend^(39)^ and Rogers’ Diffusion of Innovations model^(27)^, highlighting the importance of grounding technology-mediated programs in sound pedagogical principles. The use of technology with educational intent is essential, requiring pedagogical support in the structures, actors, and teaching strategies^(53)^. In an e-activities framework, the design of online activities with intentional pedagogical strategies is crucial to promote engagement and active participation, going beyond the simple transfer of traditional practices to digital formats^(54)^. Similarly, effective online education requires structuring courses based on pedagogical theories that facilitate meaningful interactions^(55)^, such as the Community of Inquiry model^(56)^, ensuring that technology-enhanced programs are more than just innovative - they are pedagogically sound. Online educational programs are seen as innovative and complementary approaches^(57)^, and blended learning, in particular, has the potential to increase the effectiveness and efficiency of educational activities^(58)^.

These programs were strongly influenced by a hands-on approach, incorporating guidelines based on recommended directives. It was also observed that the influence of nursing theoretical models, such as the student-centered framework of the 21st Century School Nursing Practice™^(47)^ and Benner’s model^(37)^, can be crucial for structuring nursing practice and enriching the educational experience^(59)^.

The definition of CSHCN is not limited to the presence of a clinical diagnosis but rather encompasses the consequences that a condition can generate across various domains, making the concept broader and more inclusive^(60)^. However, the programs identified in this research review tend to focus on specific health conditions. The analyzed programs tend to prioritize conditions highlighted in the epidemiological profile of children requiring urgent interventions, such as diabetes and asthma, likely due to the high risk of serious or fatal outcomes^(61–63)^.

Training programs were fundamental in all collected studies, promoting educational interventions that provided nurses with the knowledge, skills, and confidence needed to deliver quality care. These findings confirm the positive impact of continuous education on maintaining high standards of nursing care^(64)^. Ongoing training is essential for the effective management of special health needs in the school environment, and this should be adapted to the specific needs of nurses^(65)^.

The teaching strategies applied in the studies ranged from PowerPoint modules to interactive discussion forums, promoting both autonomous and collaborative learning. The need for environments that facilitate interaction among nurses is emphasized, as well as the flexibility provided by digital technologies. Additionally, e-learning has emerged as an effective alternative for continuing education in nursing, demonstrating positive results in terms of satisfaction, flexibility, and content relevance^(45,66,67)^.

All the analyzed studies emphasized the importance of detailing how the assessment of knowledge, skill development, self-efficacy, and professional confidence of nurses occur, integrating assessment practices into training planning. Competency-based training aims to improve population’s health outcomes^(68)^. The results of this review indicate that such programs can strengthen the self-efficacy and confidence of school nurses^(69)^. Self-efficacy influences the perception of the ability to change behaviors, which is crucial for the acquisition of clinical skills^(70)^. Although it was not mentioned, Kirkpatrick evaluation model is widely used to measure training effectiveness through four levels^(71)^.

The results show that the evaluations conducted predominantly focused on the summative component, neglecting formative assessment, which is crucial for monitoring the learning process during the design and development of training. Furthermore, the mere incorporation of technologies into assessment does not ensure an effective transformation of evaluation methods^(72)^. This underscores the urgency for adaptations in assessment in response to changes in educational environments resulting from the migration to digital spaces^(73)^. It is also essential to explore new paradigms of assessment and self-assessment in technology-mediated training models, recognizing and valuing the diversity of digital assessment methods. To this end, it is recommended to integrate assessment tasks and activities that simulate real-life situations^(72)^. In this context, the implementation of continuous assessment strengthens the structure of the evaluation model, emphasizing its formative function and promoting the development of learners by placing them at the center of the educational process.

This review had limitations, such as only searching for studies in English, Portuguese, or Spanish. In spite of these limitations, it identified a knowledge gap, providing insights into future research and suggesting an approach for developing technology-mediated training programs focusing on special health needs in a school context.

## CONCLUSION

This scoping review revealed significant methodological and practical implications for the development of training programs aimed at school teams, with a focus on special health needs. The use of multiple digital resources enriches the training, fostering both individual and collaborative learning. Looking ahead, it is crucial to address the lack of a solid pedagogical framework for digital environments, the need for further research into the various domains associated with special health needs, particularly those involving support from interdisciplinary school teams, and for training adapted to digital formats, incorporating consistent and suitable formative evaluation tools.

## References

[1] Cygan H, Tribbia C, Tully J. (2020). School Health Policy Implementation: facilitators and challenges. J Sch Nurs.

[2] Kim S, Lee H, Lee H, Loan BTT, Huyen LTT, Huong NTT. (2020). Prioritizing training needs of school health staff: the example of Vietnam. Int J Environ Res Public Health.

[3] American Nurses Association. (2022). Nursing: scope and standards of pratice..

[4] World Health Organization. (2021). Guideline on school health services [Internet].. https://www.who.int/publications/i/item/9789240029392.

[5] McPherson M, Arango P, Fox H, Lauver C, McManus M, Newacheck PW (1998). A new definition of children with special health care needs. Pediatrics.

[6] Centers for Disease Control and Prevention. (2023). School nurses help keep students healthy [Internet].. https://www.cdc.gov/healthyschools/features/school_nurse.htm.

[7] Bethell C, Forrest C, Stumbo S, Gombojav N, Carle A, Irwin C. (2012). Factors promoting or potentially impeding school success: disparities and state variations for children with special health care needs. Matern Child Health J.

[8] Houtrow A, Jones J, Ghandour R, Strickland B, Newacheck P. (2012). Participation of children with special health care needs in school and the community. Acad Pediatr.

[9] Forrest CB, Bevans KB, Riley AW, Crespo R, Louis TA. (2011). School outcomes of children with special health care needs. Pediatrics.

[10] World Health Organization. (2022). Roadmap for implementation of health promoting Schools in the South-East Asia Region [Internet].. https://www.who.int/publications/i/item/9789290209799.

[11] UNESCO. (2022). Education for sustainable development goals: learming objectives [Internet].. UNESCO.

[12] Baltag V, Pachyna A, Hall J. (2015). Global overview of school health services: data from 102 countries. Health Behav Policy Rev.

[13] American Academy of Pediatrics. (2022). School health services [Internet].. AAP.

[14] Argyri T, Zoulias E, Liaskos J, Mantas J. (2022). Use of scratch as ICT educational tool in health. Stud Health Technol Inform.

[15] European Comission. (2021). Plano de ação para a educação digital (2021–2027) [Internet].. European Comission.

[16] Frenk J, Chen LC, Chandran L, Groff EOH, King R, Meleis A (2022). Challenges and opportunities for educating health professionals after the COVID-19 pandemic. Lancet.

[17] Plummer ML, Chan A, Kohl K, Taylor AB, Baltag V, Saewyc E (2021). Results of a Global Survey of Experts to Categorize the Suitability of Interventions for Inclusion in School Health Services. J Adolesc Health.

[18] Centers for Disease Control and Prevention. (2024). About training development [Internet].. https://www.cdc.gov/training-development/php/about/index.html.

[19] Goodyear P. (2023). An education in educational technology. Australas J Educ Technol.

[20] Peters MD, Godfrey C, McInerney P, Baldini Soares C, Khalil H, Parker D., Aromataris E, Munn Z (2020). Joanna Briggs Institute Reviewer’s Manual..

[21] Pollock D, Peters MDJ, Khalil H, McInerney P, Alexander L, Tricco AC (2023). Recommendations for the extraction, analysis, and presentation of results in scoping reviews. JBI Evid Synth.

[22] Tricco AC, Lillie E, Zarin W, O’Brien KK, Colquhoun H, Levac D (2018). PRISMA Extension for Scoping Reviews (PRISMA-ScR): checklist and explanation. Ann Intern Med.

[23] Page MJ, McKenzie JE, Bossuyt PM, Boutron I, Hoffmann TC, Mulrow CD (2021). The PRISMA 2020 statement: an updated guideline for reporting systematic reviews. PLoS Med.

[24] Bachman JA, Hsueh KH. (2008). Evaluation of online education about diabetes management in the school setting. J Sch Nurs.

[25] Missouri Department of Health and Senior Services. (2006). Diabetes management in the school setting: a resource guide for school nurses [Internet].. Missouri Department of Health and Senior Services.

[26] National Institute of Diabetes and Digestive and Kidney Diseases (2003). Helping the student with diabetes succeed: a guide for school personnel [Internet]. http://ndep.nih.gov/diabetes/pubs/Youth_NDEPSchoolGuide.pdf.

[27] Rogers M. (2003). Diffusion of Innovations..

[28] Elgie R, Sapien R, Fullerton L, Moore B. (2010). School nurse online emergency preparedness training: an analysis of knowledge, skills, and confidence. J Sch Nurs.

[29] Russ-Eft D. (2004). Toward a meta-theory of learning and performance. In: Institute of Education Sciences. Toward a meta-theory of learning and performance [Internet].. http://www.eric.ed.gov/ERICDocs/data/ericdocs2sql/content_storage_01/0000019b/80/1b/da/6d.pdf.

[30] Putman-Casdorph H, Pinto S. (2011). Preliminary Testing of an Asthma Distance Education Program (ADEP) for School Nurses in Appalachia. J Sch Nurs.

[31] National Asthma Education and Prevention Program EPR 3 (2007). Guidelines for the Diagnosis and Management of Asthma [Internet].. NHLBI.

[32] Cicutto L, Gleason M, Haas-Howard C, Jenkins-Nygren L, Labonde S, Patrick K. (2017). Competency-based framework and continuing education for preparing a skilled school health workforce for asthma care: the colorado experience. J Sch Nurs.

[33] National Asthma Educator Certification Board (2015). Certified Asthma Educator (AE-C) Handbook [Internet].. NAECB.

[34] Erickson CD, Splett PL, Mullet SS, Heiman MB. (2006). The Healthy Learner Model for Student Chronic Condition Management--Part I. J Sch Nurs.

[35] Erickson CD, Splett PL, Mullett SS, Jensen C, Belseth SB. (2006). The Healthy Learner Model for student chronic condition management -- part II: the asthma initiative. J Sch Nurs.

[36] Blackmon-Jones L. (2017). A strategy to promote successful transition to school nursing. NASN Sch Nurse.

[37] Benner PE. (1984). From novice to expert: excellence and power in clinical nursing practice..

[38] Rhodes D, Visker J, Larson K, Cox C. (2019). Rapid E-Learning for professional development in school-based diabetes management. Nurse Educ Pract.

[39] Salyers V, Carter L, Cairns N, Durrer L. (2014). The use of scaffolding and interactive learning strategies in online courses for working nurses: implications for adult and online education. CJUCE.

[40] Shimasaki S, Brunner Nii P, Davis L, Bishop E, Berget C, Perreault C (2021). A School Nurse Application of the ECHO Model. J Sch Nurs.

[41] National Association of School Nurses. (2016). Framework for 21st Century School Nursing Practice: National Association of School Nurses. NASN Sch Nurse.

[42] American Diabetes Association. (2018). 12. Children and Adolescents: Standards of Medical Care in Diabetes-2018.. Diabetes Care.

[43] American Diabetes Association (2021). Training Resources for School Staff [Internet].. American Diabetes Association.

[44] Council on School Health. (2016). Role of the school nurse in providing school health services. Pediatrics.

[45] Anderson LS, Enge KJ. (2012). Education and information for practicing school nurses: which technology-supported resources meet their needs?. J Sch Nurs.

[46] National Association of School Nurses (2023). Our history [Internet].. NASN.

[47] Davis D, Maughan ED, White KA, Slota M. (2021). School Nursing for the 21st Century: Assessing Scope of Practice in the Current Workforce. J Sch Nurs.

[48] Health Resources and Services Administration (2022). Maternal and Child Health Bureau. Children and youth with special health care needs: NSCH Data Brief [Internet].. MCHB.

[49] Vilaça T, Darlington E, Velasco MJM, Martinis O, Masson J. (2019). SHE School manual 2.0. A methodological guidebook to become a health promoting school [Internet].. https://hdl.handle.net/1822/71403.

[50] Fonseca L, Sena BF, Crossley N, Lopez-Jaramillo C, Koenen K, Bressan RA (2021). Diversity matters: opportunities in the study of the genetics of psychotic disorders in low- and middle-income countries in Latin America.. Braz J Psychiatry..

[51] Aravena Castillo FHP. (2018). Systematic review of research on educational leadership and management in Latin America, 1991-2017. Educ Manage Adm Leadersh.

[52] Silva J. (2017). Un modelo pedagógico virtual centrado en las e-actividades. RED.

[53] Moreira JA. (2018). authors. Educação a distância: dimensões da pesquisa, da mediação e da formação..

[54] Salmon G. (2013). E-tivities: the key to active online learning..

[55] McKenna G. (2009). Reviews: theory and practice of online learning. Int J Inf Manage.

[56] Garrison DR, Anderson T, Archer W. (2000). Critical inquiry in a text-based environment: computer conferencing in higher education. Internet High Educ.

[57] Schuler MS, Tyo MB, Barnett K. (2021). Nursing student perceptions of required online educational programs utilized outside the classroom. Nurse Educ Today.

[58] Garrison DR, Kanuka H. (2004). Blended learning: uncovering its transformative potential in higher education. Internet High Educ.

[59] Alligood MR. (2018). Nursing theorists and their work.. Louis, MO: Mosby/Elsevier.

[60] Bethell CD, Blumberg SJ, Stein REK, Strickland B, Robertson J, Newacheck PW. (2015). Taking Stock of the CSHCN Screener: a review of common questions and current reflections. Acad Pediatr.

[61] Ghandour RM, Hirai AH, Kenney MK. (2022). Children and youth with special health care needs: a profile. Pediatrics.

[62] Allen K, Henselman K, Laird B, Quiñones A, Reutzel T. (2012). Potential Life-Threatening Events in Schools Involving Rescue Inhalers, Epinephrine Autoinjectors, and Glucagon Delivery Devices: Reports from School Nurses. J Sch Nurs.

[63] Greene GM. (2019). Perceived confidence levels of NC school nurses with emergency preparedness [Internet].. Gardner-Webb University.

[64] Mlambo M, Silén C, McGrath C. (2021). Lifelong learning and nurses’ continuing professional development, a metasynthesis of the literature. BMC Nurs.

[65] Shin EM, Roh YS. (2020). A school nurse competency framework for continuing education. Healthcare (Basel).

[66] Amante L, Oliveira I, Pereira A. (2017). Cultura da Avaliação e Contextos Digitais de Aprendizagem: O modelo PrACT.. Rev Docência e Cibercultura..

[67] Rouleau G, Gagnon MP, Côté J, Payne-Gagnon J, Hudson E, Dubois CA (2019). Effects of E-learning in a continuing education context on nursing care: systematic review of systematic qualitative, quantitative, and mixed-studies reviews. J Med Internet Res.

[68] International Council of Nursing (2022). The international council of nurses welcomes WHO’s new competency framework in world health worker week [Internet]. https://www.icn.ch/news/international-council-nurses-welcomes-whos-new-competency-framework-world-health-worker-week.

[69] Austin JK, Kakacek JR, Carr D. (2010). Impact of training program on school nurses’ confidence levels in managing and supporting students with epilepsy and seizures. J Sch Nurs.

[70] Shorey S, Lopez V., Haugan G, Eriksson M (2021). Self-efficacy in a nursing context. In:. Health promotion in health care–vital theories and research. Cham: Springer.

[71] Kirkpatrick DL, Kirkpatrick JD. (2007). Implementing the four levels..

[72] Amante L, Oliveira I. (2019). Modelo pedagógico virtual. Avaliação e feedback: desafios atuais [Internet].. http://hdl.handle.net/10400.2/8419%0A.

[73] Moreira JA, Henriques S, Barros DMV, Goulão F, Caeiro D. (2020). Educação digital em rede: princípios para o design pedagógico em tempos de pandemia..

