# Supplementary material for: Technology-mediated training programs for school health teams on special health care needs: a scoping review
Source: Rev Esc Enferm USP. 2025 May 9;59:e20240328. doi: 10.1590/1980-220X-REEUSP-2024-0328en (PMC12064086; doi:10.1590/1980-220X-REEUSP-2024-0328en)
Supplement: Translation

# Translation: Programas de capacitación mediados por tecnología para equipos de salud escolar sobre necesidades especiales de atención de salud: una revisión del alcance

**DOI:** 10.1590/1980-220X-REEUSP-2024-0328es

**Authors:** Maria do Céu Coelho Monteiro Pires, Maria do Céu Aguiar Barbieri - Figueiredo, Daniela Filipa Batista Cardoso, Filipa Margarida Duque, Maria Sandra Tricas-Sauras, Mirko Prosen, Eva Guilherme Menino

**Affiliations:** 1 Universidade do Porto, Instituto de Ciências Biomédicas Abel Salazar, Porto, Portugal.; 2 Escola Superior de Saúde da Cruz Vermelha Portuguesa - Lisboa, Lisboa, Portugal.; 3 Universidad de Huelva, Facultad Enfermería, Huelva, Espanha.; 4 Centro Português para a Prática Baseada na Evidência, Unidade de Investigação em Ciências da Saúde: Enfermagem, Escola Superior de Enfermagem de Coimbra, Coimbra, Portugal.; 5 Escola Superior de Enfermagem de Coimbra, Unidade de Investigação em Ciências da Saúde: Enfermagem, Coimbra, Portugal.; 6 Université Libre de Bruxelles, École de Santé Publique, Centre de Recherche sur les Approches Sociales de la Santé, Bruxelles, Belgique.; 7 University of Primorska, Faculty of Health Sciences, Department of Nursing, Izola, Slovenia.; 8 Center for Innovative Care and Health Technology, Escola Superior de Saúde, Instituto Politécnico de Leiria, Leiria, Portugal

**Keywords:** Servicios de Salud Escolar, Salud Infantil, Capacitación Profesional, Tecnología Educacional, Servicios de Enfermería Escolar

## Abstract

**Objetivo:**

Mapear programas de formación mediados por tecnología para equipos de salud escolar que aborden necesidades especiales de salud en el entorno escolar e identificar sus características.

**Método:**

La revisión siguió la metodología del JBI. Se buscaron literatura publicada y gris en ocho bases de datos. Se incluyeron estudios publicados en portugués, inglés o español desde el año 2000 para identificar los programas de formación emergentes.

**Resultados:**

De los 1.106 estudios identificados, 29 fueron revisados en su totalidad y ocho fueron incluidos en el análisis final. Todos los estudios fueron realizados en los Estados Unidos de América. Los temas del programa incluyeron condiciones de salud crónicas como diabetes, asma, procedimientos, así como situaciones de emergencia, todos dirigidos a enfermeros escolares y basados en un marco conceptual o pedagógico. Los programas incluyeron módulos temáticos de diversa duración y ofrecieron formación en línea e híbrida a través de diversos recursos educativos digitales.

**Conclusiones:**

Los programas se centraron en las condiciones de salud crónicas y evaluaron el conocimiento, el desarrollo de habilidades, la autoeficacia y la confianza de los profesionales; pocos estudios proporcionaron una exploración detallada de los modelos pedagógicos subyacentes y no utilizaron una evaluación formativa.

## INTRODUCCIÓN

La escuela desempeña un papel fundamental en la vida de los niños y los jóvenes. Además de promover la educación, también es responsable de promover la salud y el bienestar, ya que los alumnos pasan gran parte de su tiempo en el entorno escolar^(1–4)^. La Academia Americana de Pediatría (AAP) define a los niños y jóvenes con necesidades especiales de atención sanitaria (CSHCN en la sigla en inglés) como aquellos que padecen o corren el riesgo de padecer enfermedades crónicas que requieren atención sanitaria y servicios especializados^(5)^. Estos niños se enfrentan a desventajas en el entorno escolar, con un mayor riesgo de exclusión de las actividades educativas y sociales, lo que puede repercutir negativamente en su rendimiento académico^(6, 7, 8, 9)^. Por lo tanto, la integración de la salud y la educación es esencial para crear un ciclo de salud universal, que garantice el acceso a una educación inclusiva y de calidad para todos los niños, independientemente de sus condiciones de salud^(10,11)^.

Según la Organización Mundial de la Salud (OMS), los servicios de salud escolar son prestados por profesionales de la salud dentro de las escuelas o en colaboración con servicios externos^(4)^. Aunque la composición de los equipos de salud escolar no aparece muy detallada en la bibliografía, suele incluir enfermeras, médicos y psicólogos^(3,4,12)^. En particular, las enfermeras escolares, que requieren formación especializada y desarrollo profesional continuo, desempeñan un papel esencial en el apoyo diario a las necesidades de salud de los estudiantes^(2,3,13)^.

La transformación digital ha sido un aliado crucial en este proceso, facilitando el acceso a la información y formación en salud, y explorando nuevas herramientas que hagan la educación más inclusiva y adaptada a la era digital^(14,15)^. La pandemia de COVID-19 reforzó aún más esta importancia, acelerando la incorporación de tecnologías para promover el aprendizaje^(15,16)^.

La mayoría de las intervenciones consideradas esenciales en los servicios de salud escolar se refieren a la promoción de la salud y la educación sanitaria, frente a las relacionadas con la inclusión de los CSHCN^(17)^. Además, uno de los desafíos más frecuentemente reportados para los servicios de salud escolar es la falta de oportunidades de capacitación y desarrollo profesional continuo^(4)^.

Los programas de capacitación son conjuntos estructurados de actividades interrelacionadas diseñadas para desarrollar conocimientos, actitudes y habilidades en áreas específicas^(18)^. Estos programas incluyen intervenciones educativas, en este caso mediadas por tecnologías, que fomentan el aprendizaje y el desarrollo de capacidades, mejorando el rendimiento y las competencias de los participantes.

En este estudio, el término tecnología se refiere a la tecnología educativa como la aplicación de herramientas y recursos tecnológicos para mejorar la enseñanza y el aprendizaje. Abarca el uso de diversas herramientas digitales, software y dispositivos para mejorar los resultados y las experiencias educativas^(19)^.

La literatura es escasa en relación con el proceso pedagógico y el marco conceptual de la formación, específicamente en lo que se refiere a la formación mediada por tecnología. Tampoco están bien explicitadas las principales características de estos programas ni la relación entre los resultados del aprendizaje y las herramientas de evaluación utilizadas.

Esta revisión de alcance tuvo como objetivo mapear los programas de formación mediados por tecnología para los equipos de salud escolar, centrándose en las necesidades especiales de atención sanitaria en el ámbito escolar. Las preguntas específicas de la revisión fueron: ¿Qué modelos pedagógicos, teorías o principios conceptuales se han utilizado para diseñar estos programas de formación? ¿Abordan estos programas de formación condiciones sanitarias específicas? ¿Cuál es la organización del proceso de formación (objetivos de aprendizaje, programa, calendario, modalidad, tipo de tecnología y participantes) y las estrategias de enseñanza y aprendizaje? ¿Qué resultados de aprendizaje se evaluaron y qué instrumentos se utilizaron para evaluar los objetivos de aprendizaje deseados?

La razón específica para elegir una revisión de alcance es que su objetivo es mapear la evidencia existente subyacente a un área de investigación, identificar lagunas en la evidencia existente, y construir un ejercicio precursor que justifique e informe la realización de una revisión sistemática de la literatura científica^(20)^.

## MÉTODO

Una búsqueda realizada el 13 de septiembre de 2021 en MEDLINE (PubMed) y en el JBI Evidence Synthesis, reveló que no hay ninguna revisión bibliográfica disponible o en desarrollo sobre programas de formación mediados por tecnología para equipos de salud escolar centrados en necesidades especiales de salud. Por lo tanto, se deben identificar oportunidades de formación mediada por tecnología para los equipos de salud escolar para que desarrollen habilidades en su práctica y encontrar lagunas para futuras investigaciones en el campo de la enfermería escolar.

### Diseño del Estudio

Se siguió la metodología del JBI para las revisiones de alcance, y las recomendaciones de la Lista de verificación de los Elementos de Información Preferidos para Revisiones Sistemáticas y Metaanálisis (PRISMA-ScR)^(21,22)^asociada al Diagrama de flujo PRISMA 2020^(23)^. No se evaluó especialmente la calidad de los estudios por tratarse de una revisión de alcance. El protocolo de revisión se llevó a cabo y se registró en el Open Science Framework (OSF) con el título de la revisión y el DOI 10.17605/OSF.IO/53Z89.

### Fuentes de Datos y Estrategia de Investigación

La pregunta de revisión se formuló de acuerdo con el marco PCC, siendo la Población (P) los equipos de salud escolares, el Concepto (C) se centró en los programas de formación mediados por tecnología que abordan las necesidades especiales de atención sanitaria, y el Contexto (C) abarcó cualquier lugar donde pueda tener lugar la formación. Los tipos de fuentes de evidencia incluían estudios primarios que utilizaban métodos cualitativos, cuantitativos o mixtos, así como revisiones sistemáticas. La revisión se restringió a estudios publicados en portugués, inglés o español a partir del año 2000.

La revisión incluyó estudios que involucraron a profesionales de la salud de equipos de salud escolar, particularmente aquellos calificados en promoción de la salud y apoyo a CSHCN. También incluyó programas de capacitación mediados por tecnología destinados a mejorar las habilidades de los equipos de salud escolar en el manejo de CSHCN y condiciones crónicas de salud, a saber, intervenciones educativas, tales como sesiones, seminarios, conferencias y desarrollo profesional continuo, diseñados específicamente para profesionales de la salud en el contexto escolar. El contexto incluía cualquier entorno de formación, y el periodo cronológico seleccionado refleja los avances tecnológicos de principios del siglo XXI, destacando la evolución de la tecnología en la educación y la formación. Se excluyeron los estudios dirigidos a profesionales no sanitarios, como profesores, y los estudios que carecían de una identificación clara de los programas de formación.

Se utilizó una estrategia de búsqueda en tres etapas. En primer lugar, se realizó una búsqueda inicial en MEDLINE (a través de PubMed), CINAHL (a través de EBSCO) utilizando palabras clave generales, como «School Health» AND technolog* AND (lifelong or Continuing). Los resultados de esta búsqueda inicial se analizaron para identificar otras palabras clave y términos de índice que se utilizarían en la estrategia de búsqueda final. Estos términos se organizaron en una cuadrícula lógica que sirvió de base para realizar la segunda fase de la búsqueda. En la segunda fase, se realizaron búsquedas en cinco bases de datos: MEDLINE (vía PubMed), CINAHL (vía EBSCO), SCOPUS, Psychology and Behavioral Sciences Collection (vía EBSCO) y LILACS, aplicando las palabras clave y términos indexados identificados previamente y adaptados para cada base de datos incluida.

La búsqueda de literatura gris se realizó en el *Repositório Científico de Acesso Aberto de Portugal [Repositorio Científico de Acceso Abierto de* Portugal] (RCAAP), *Catálogo de Teses & Dissertações [Catálogo de Tesis y Disertaciones* ] (CAPES) y Mednar. Además, se realizaron búsquedas en tres sitios web oficiales y gubernamentales relacionados con el tema, en concreto, los Centros para el Control y la Prevención de Enfermedades (CDC en la sigla en inglés) - Escuela de Salud, la Asociación Nacional de Enfermeras de Programas Escolares (NASN en la sigla en inglés) y las Enfermeras de Programas Escolares de Nueva Zelanda (NZ+SN en la sigla en inglés). En la tercera fase, se buscaron estudios adicionales en la lista de referencias de los estudios seleccionados, así como en la literatura gris. La estrategia de búsqueda completa para cada base de datos y las fuentes de literatura gris se detallan en el Cuadro 1.

**Cuadro 1:** Estrategias de búsqueda de la revisión de alcance – Coimbra, Portugal, 2021–2022.

MEDLINE vía PubMed: Búsqueda en 11 de noviembre de 2021		
ID	Estrategia de búsqueda	
#19	Search: (((“school nurses”[Title/Abstract] OR “School Health”[Title/Abstract] OR “School-Based Health”[Title/Abstract] OR “School Based Services”[Title/Abstract] OR “School Based Service”[Title/Abstract] OR “School-Based Service”[Title/Abstract] OR “School-Based Services”[Title/Abstract] OR “School Based Health”[Title/Abstract] OR “school nurse”[Title/Abstract]) OR (“School Health Services”[Mesh])) AND ((Continuing[Title/Abstract] OR “lifelong learning”[Title/Abstract] OR “professional development”[Title/Abstract]) OR (“Education, Continuing”[Mesh]))) AND (((((((Technolog*[Title/Abstract] OR Computer*[Title/Abstract] OR Online[Title/Abstract] OR Smartphone*[Title/Abstract] OR Electronic*[Title/Abstract] OR Virtual[Title/Abstract] OR Augmented[Title/Abstract] OR Mobile[Title/Abstract] OR Gamification[Title/Abstract] OR Platform*[Title/Abstract] OR Internet[Title/Abstract] OR e-learn*[Title/Abstract] OR MOOC[Title/Abstract] OR Distance[Title/Abstract] OR Hybrid[Title/Abstract] OR Digital[Title/Abstract] OR Blend*[Title/Abstract]) OR (“Education, Distance”[Mesh])) OR (“Computer User Training”[Mesh])) OR (“Computer Simulation”[Mesh])) OR (“Computer-Assisted Instruction”[Mesh])) OR (“Educational Technology”[Mesh])) OR (“Digital Technology”[Mesh])) Filters: English, Portuguese, Spanish	79
#18	Search: (((“school nurses”[Title/Abstract] OR “School Health”[Title/Abstract] OR “School-Based Health”[Title/Abstract] OR “School Based Services”[Title/Abstract] OR “School Based Service”[Title/Abstract] OR “School-Based Service”[Title/Abstract] OR “School-Based Services”[Title/Abstract] OR “School Based Health”[Title/Abstract] OR “school nurse”[Title/Abstract]) OR (“School Health Services”[Mesh])) AND ((Continuing[Title/Abstract] OR “lifelong learning”[Title/Abstract] OR “professional development”[Title/Abstract]) OR (“Education, Continuing”[Mesh]))) AND (((((((Technolog*[Title/Abstract] OR Computer*[Title/Abstract] OR Online[Title/Abstract] OR Smartphone*[Title/Abstract] OR Electronic*[Title/Abstract] OR Virtual[Title/Abstract] OR Augmented[Title/Abstract] OR Mobile[Title/Abstract] OR Gamification[Title/Abstract] OR Platform*[Title/Abstract] OR Internet[Title/Abstract] OR e-learn*[Title/Abstract] OR MOOC[Title/Abstract] OR Distance[Title/Abstract] OR Hybrid[Title/Abstract] OR Digital[Title/Abstract] OR Blend*[Title/Abstract]) OR (“Education, Distance”[Mesh])) OR (“Computer User Training”[Mesh])) OR (“Computer Simulation”[Mesh])) OR (“Computer-Assisted Instruction”[Mesh])) OR (“Educational Technology”[Mesh])) OR (“Digital Technology”[Mesh])) Filters: English, Portuguese	78
#17	Search: (((“school nurses”[Title/Abstract] OR “School Health”[Title/Abstract] OR “School-Based Health”[Title/Abstract] OR “School Based Services”[Title/Abstract] OR “School Based Service”[Title/Abstract] OR “School-Based Service”[Title/Abstract] OR “School-Based Services”[Title/Abstract] OR “School Based Health”[Title/Abstract] OR “school nurse”[Title/Abstract]) OR (“School Health Services”[Mesh])) AND ((Continuing[Title/Abstract] OR “lifelong learning”[Title/Abstract] OR “professional development”[Title/Abstract]) OR (“Education, Continuing”[Mesh]))) AND (((((((Technolog*[Title/Abstract] OR Computer*[Title/Abstract] OR Online[Title/Abstract] OR Smartphone*[Title/Abstract] OR Electronic*[Title/Abstract] OR Virtual[Title/Abstract] OR Augmented[Title/Abstract] OR Mobile[Title/Abstract] OR Gamification[Title/Abstract] OR Platform*[Title/Abstract] OR Internet[Title/Abstract] OR e-learn*[Title/Abstract] OR MOOC[Title/Abstract] OR Distance[Title/Abstract] OR Hybrid[Title/Abstract] OR Digital[Title/Abstract] OR Blend*[Title/Abstract]) OR (“Education, Distance”[Mesh])) OR (“Computer User Training”[Mesh])) OR (“Computer Simulation”[Mesh])) OR (“Computer-Assisted Instruction”[Mesh])) OR (“Educational Technology”[Mesh])) OR (“Digital Technology”[Mesh])) Filters: English	78
#16	Search: (((“school nurses”[Title/Abstract] OR “School Health”[Title/Abstract] OR “School-Based Health”[Title/Abstract] OR “School Based Services”[Title/Abstract] OR “School Based Service”[Title/Abstract] OR “School-Based Service”[Title/Abstract] OR “School-Based Services”[Title/Abstract] OR “School Based Health”[Title/Abstract] OR “school nurse”[Title/Abstract]) OR (“School Health Services”[Mesh])) AND ((Continuing[Title/Abstract] OR “lifelong learning”[Title/Abstract] OR “professional development”[Title/Abstract]) OR (“Education, Continuing”[Mesh]))) AND (((((((Technolog*[Title/Abstract] OR Computer*[Title/Abstract] OR Online[Title/Abstract] OR Smartphone*[Title/Abstract] OR Electronic*[Title/Abstract] OR Virtual[Title/Abstract] OR Augmented[Title/Abstract] OR Mobile[Title/Abstract] OR Gamification[Title/Abstract] OR Platform*[Title/Abstract] OR Internet[Title/Abstract] OR e-learn*[Title/Abstract] OR MOOC[Title/Abstract] OR Distance[Title/Abstract] OR Hybrid[Title/Abstract] OR Digital[Title/Abstract] OR Blend*[Title/Abstract]) OR (“Education, Distance”[Mesh])) OR (“Computer User Training”[Mesh])) OR (“Computer Simulation”[Mesh])) OR (“Computer-Assisted Instruction”[Mesh])) OR (“Educational Technology”[Mesh])) OR (“Digital Technology”[Mesh])) Filters: Spanish	1
#15	Search: (((“school nurses”[Title/Abstract] OR “School Health”[Title/Abstract] OR “School-Based Health”[Title/Abstract] OR “School Based Services”[Title/Abstract] OR “School Based Service”[Title/Abstract] OR “School-Based Service”[Title/Abstract] OR “School-Based Services”[Title/Abstract] OR “School Based Health”[Title/Abstract] OR “school nurse”[Title/Abstract]) OR (“School Health Services”[Mesh])) AND ((Continuing[Title/Abstract] OR “lifelong learning”[Title/Abstract] OR “professional development”[Title/Abstract]) OR (“Education, Continuing”[Mesh]))) AND (((((((Technolog*[Title/Abstract] OR Computer*[Title/Abstract] OR Online[Title/Abstract] OR Smartphone*[Title/Abstract] OR Electronic*[Title/Abstract] OR Virtual[Title/Abstract] OR Augmented[Title/Abstract] OR Mobile[Title/Abstract] OR Gamification[Title/Abstract] OR Platform*[Title/Abstract] OR Internet[Title/Abstract] OR e-learn*[Title/Abstract] OR MOOC[Title/Abstract] OR Distance[Title/Abstract] OR Hybrid[Title/Abstract] OR Digital[Title/Abstract] OR Blend*[Title/Abstract]) OR (“Education, Distance”[Mesh])) OR (“Computer User Training”[Mesh])) OR (“Computer Simulation”[Mesh])) OR (“Computer-Assisted Instruction”[Mesh])) OR (“Educational Technology”[Mesh])) OR (“Digital Technology”[Mesh]))	79
#14	Search: ((((((Technolog*[Title/Abstract] OR Computer*[Title/Abstract] OR Online[Title/Abstract] OR Smartphone*[Title/Abstract] OR Electronic*[Title/Abstract] OR Virtual[Title/Abstract] OR Augmented[Title/Abstract] OR Mobile[Title/Abstract] OR Gamification[Title/Abstract] OR Platform*[Title/Abstract] OR Internet[Title/Abstract] OR e-learn*[Title/Abstract] OR MOOC[Title/Abstract] OR Distance[Title/Abstract] OR Hybrid[Title/Abstract] OR Digital[Title/Abstract] OR Blend*[Title/Abstract]) OR (“Education, Distance”[Mesh])) OR (“Computer User Training”[Mesh])) OR (“Computer Simulation”[Mesh])) OR (“Computer-Assisted Instruction”[Mesh])) OR (“Educational Technology”[Mesh])) OR (“Digital Technology”[Mesh])	2,493,775
#13	Search: “Digital Technology”[Mesh] Sort by: Most Recent	349
#12	Search: “Educational Technology”[Mesh] Sort by: Most Recent	112,531
#11	Search: “Computer-Assisted Instruction”[Mesh] Sort by: Most Recent	12,258
#10	Search: “Computer Simulation”[Mesh] Sort by: Most Recent	265,374
#9	Search: “Computer User Training”[Mesh] Sort by: Most Recent	2,041
#8	Search: “Education, Distance”[Mesh] Sort by: Most Recent	5,550
#7	Search: Technolog*[Title/Abstract] OR Computer*[Title/Abstract] OR Online[Title/Abstract] OR Smartphone*[Title/Abstract] OR Electronic*[Title/Abstract] OR Virtual[Title/Abstract] OR Augmented[Title/Abstract] OR Mobile[Title/Abstract] OR Gamification[Title/Abstract] OR Platform*[Title/Abstract] OR Internet[Title/Abstract] OR e-learn*[Title/Abstract] OR MOOC[Title/Abstract] OR Distance[Title/Abstract] OR Hybrid[Title/Abstract] OR Digital[Title/Abstract] OR Blend*[Title/Abstract]	2,205,126
#6	Search: (Continuing [Title/Abstract] OR “lifelong learning”[Title/Abstract] OR “professional development”[Title/Abstract]) OR (“Education, Continuing”[Mesh])	139,978
#5	Search: (“school nurses”[Title/Abstract] OR “School Health”[Title/Abstract] OR “School-Based Health”[Title/Abstract] OR “School Based Services”[Title/Abstract] OR “School Based Service”[Title/Abstract] OR “School-Based Service”[Title/Abstract] OR “School-Based Services”[Title/Abstract] OR “School Based Health”[Title/Abstract] OR “school nurse”[Title/Abstract]) OR (“School Health Services”[Mesh])	28,076
#4	Search: “Education, Continuing”[Mesh] Sort by: Most Recent	62,175
#3	Search: Continuing [Title/Abstract] OR “lifelong learning”[Title/Abstract] OR “professional development”[Title/Abstract]	91,552
#2	Search: “School Health Services”[Mesh] Sort by: Most Recent	23,930
#1	Search: “school nurses”[Title/Abstract] OR “School Health”[Title/Abstract] OR “School-Based Health”[Title/Abstract] OR “School Based Services”[Title/Abstract] OR “School Based Service”[Title/Abstract] OR “School-Based Service”[Title/Abstract] OR “School-Based Services”[Title/Abstract] OR “School Based Health”[Title/Abstract] OR “school nurse”[Title/Abstract]	11,013
**SCOPUS:** Búsqueda en 11 de noviembre de 2021		
Estrategia de búsqueda		
( TITLE-ABS-KEY ( “school nurses” OR “School Health” OR “School-Based Health” OR “School Based Services” OR “School Based Service” OR “School-Based Service” OR “School-Based Services” OR “School Based Health” OR “school nurse” ) AND TITLE-ABS-KEY ( continuing OR “lifelong learning” OR “professional development” ) AND TITLE-ABS-KEY ( technolog* OR computer* OR online OR smartphone* OR electronic* OR virtual OR augmented OR mobile OR gamification OR platform* OR internet OR e-learn* OR mooc OR distance OR hybrid OR digital OR blend* ) )		118
**Psychology & Behavioral Sciences Collection via EBSCOhost:** Búsqueda en 11 de noviembre de 2021		
Estrategia de búsqueda		
S1 AND S2 AND S3		4
TI ( Technolog* OR Computer* OR Online OR Smartphone* OR Electronic* OR Virtual OR Augmented OR Mobile OR Gamification OR Platform* OR Internet OR e-learn* OR MOOC OR Distance OR Hybrid OR Digital OR Blend* ) OR AB ( Technolog* OR Computer* OR Online OR Smartphone* OR Electronic* OR Virtual OR Augmented OR Mobile OR Gamification OR Platform* OR Internet OR e-learn* OR MOOC OR Distance OR Hybrid OR Digital OR Blend* )		93,277
TI (Continuing OR “lifelong learning” OR “professional development”) OR AB (Continuing OR “lifelong learning” OR “professional development”)		8,284
TI ( “school nurses” OR “School Health” OR “School-Based Health” OR “School Based Services” OR “School Based Service” OR “School-Based Service” OR “School-Based Services” OR “School Based Health” OR “school nurse” ) OR AB ( “school nurses” OR “School Health” OR “School-Based Health” OR “School Based Services” OR “School Based Service” OR “School-Based Service” OR “School-Based Services” OR “School Based Health” OR “school nurse” )		1,386
**CHINAL** Completada vía EBSCOhost: Búsqueda en el 6 de noviembre de 2021		
Estrategia de búsqueda		
S9 AND S18		72
S10 OR S11 OR S12 OR S13 OR S14 OR S15 OR S16 OR S17		529,504
(MH “Digital Technology”)		520
(MH “Educational Technology”)		2,270
(MH “Programmed Instruction+”)		8,693
(MH “Gamification”)		108
(MH “Computer Simulation+”)		24,615
(MH “Computer User Training”)		790
(MH “Education, Non-Traditional+”)		12,192
TI ( Technolog* OR Computer* OR Online OR Smartphone* OR Electronic* OR Virtual OR Augmented OR Mobile OR Gamification OR Platform* OR Internet OR e-learn* OR MOOC OR Distance OR Hybrid OR Digital OR Blend* ) OR AB ( Technolog* OR Computer* OR Online OR Smartphone* OR Electronic* OR Virtual OR Augmented OR Mobile OR Gamification OR Platform* OR Internet OR e-learn* OR MOOC OR Distance OR Hybrid OR Digital OR Blend* )		506,762
S5 AND S8		572
S6 OR S7		77,866
(MH “Education, Continuing+”)		36,102
TI (Continuing OR “lifelong learning” OR “professional development”) OR AB (Continuing OR “lifelong learning” OR “professional development”)		50,779
S1 OR S2 OR S3 OR S4		28,980
(MH “School Health Services+”)		24,084
(MH “School Health”)		4,067
(MH “National Association of School Nurses”)		557
TI ( “school nurses” OR “School Health” OR “School-Based Health” OR “School Based Services” OR “School Based Service” OR “School-Based Service” OR “School-Based Services” OR “School Based Health” OR “school nurse” ) OR AB ( “school nurses” OR “School Health” OR “School-Based Health” OR “School Based Services” OR “School Based Service” OR “School-Based Service” OR “School-Based Services” OR “School Based Health” OR “school nurse” )		7,426
**LILACS:** Búsqueda en 21 de marzo de 2022		
ID	Estrategia de búsqueda	
	(“school health”) AND (continuing)	25
**RCAAP:** Búsqueda en 21 de marzo de 2022		
ID	Estrategia de búsqueda	
	“school health”	52
**CAPES:** Búsqueda en 21 de marzo de 2022		
ID	Estrategia de búsqueda	
	“school health” AND continuing	23
**Mednar:** Búsqueda en 21 de marzo de 2022		
ID	Estrategia de búsqueda	
	“school health” AND continuing AND technology	733

Los duplicados se eliminaron con el programa Mendeley Reference Manager. Se utilizó el software Rayyan Qatar Computing Research Institute (Rayyan QCRI) para ayudar a archivar, organizar y seleccionar los artículos por título y resumen.

### Selección de Estudios y Extracción de Datos

El proceso de selección de estudios fue realizado de forma independiente en dos etapas por dos revisores, y un tercer revisor resolvió los conflictos. En la primera etapa se revisaron los títulos y resúmenes para excluir los artículos que no cumplían los criterios de inclusión. Si la elegibilidad de un estudio no estaba clara, se pasaba a la segunda etapa para una evaluación adicional. En esta segunda etapa, se recuperaron todos los estudios elegibles en su totalidad, se leyeron detenidamente y se analizaron. Dos revisores extrajeron los datos de forma independiente. En caso de desacuerdo, intervino un tercer revisor. Los datos extraídos incluyeron detalles sobre los autores, el año, el país, el tipo de estudio y las características de la muestra, datos sobre los programas de formación, incluida la cobertura de los participantes (número y tipo), el marco conceptual y pedagógico, los objetivos, el plan de estudios, el cronograma, la modalidad, las tecnologías utilizadas, las estrategias de enseñanza y la evaluación. Los datos se organizaron en una tabla elaborada por los autores. El formulario de extracción de datos se sometió a pruebas previas y las incoherencias se debatieron con los investigadores principales para llegar a un consenso.

### Aspectos Éticos

No fue necesaria ninguna revisión ética, ya que el estudio utilizó datos secundarios de acceso público y se respetaron todas las normas sobre derechos de autor de la investigación citada.

## RESULTADOS

De un total de 1.106 artículos potencialmente relevantes identificados inicialmente, se excluyeron 111 duplicados, así como 966 artículos en la revisión del título y el resumen, y 21 en la revisión del texto completo. Ocho artículos cumplieron los criterios de inclusión establecidos y se seleccionaron para la extracción de datos. La figura 1 ilustra el proceso de selección de los estudios, con los motivos documentados de las exclusiones.

**Figura 1:** Diagrama PRISMA de las etapas de búsqueda y selección de artículos^(23)^.

Los resultados de la revisión se sintetizaron narrativamente. Las principales características de los estudios se resumen en el Cuadro 2, seguido de un análisis que describe la relación entre estas características y los objetivos y preguntas de la revisión. Las categorías y características analizadas incluyen: la caracterización de los estudios y el marco conceptual de los programas de capacitación; la organización del proceso de capacitación (objetivos, programa, cronograma, modalidad y tecnología utilizada); las estrategias de enseñanza y aprendizaje; y los métodos de evaluación.

**Cuadro 2:** Características descriptivas detalladas de los estudios incluidos (n = 8) – Lisboa, Portugal, 2023.

Citación, país	Diseño	Objetivo	Población/muestra	Guías/marcos	Características de los programas de formación	Estrategias de enseñanza y aprendizaje	Prácticas de evaluación
1 Jean A. Bachman and Kuei-Hsiang Hsueh, 2008, Estados Unidos^(24)^.	Evaluación del programa, prueba posterior a la intervención.	Desarrollar y evaluar un programa en línea para enfermeras de programas escolares sobre el manejo de la diabetes en niños en la escuela.	N = 19 enfermeras de programas escolares	«Control de la diabetes en el entorno escolar”^(25)^. Helping the Student with Diabetes Succeed» de NIH/CDC^(26)^. Desarrollado a partir de la teoría de la difusión de las innovaciones de Rogers^(27)^.	3 lecciones a lo largo de 2-3 semanas en Blackboard® que cubren la diabetes infantil, la gestión escolar, las bombas de insulina y el papel de la enfermera escolar.	Participación en presentaciones de PowerPoint® en línea. Participación en tres foros de debate. Respuestas escritas. Moderados por el profesorado.	Incluye evaluación formativa, evaluación de los objetivos de aprendizaje y comentarios sobre las preferencias de contenido, enseñanza y formato de formación.
2 Elgie et al., 2010, Estados Unidos^(28)^.	Diseño experimental postest.	Diseño experi-mental postest. Evaluar la eficacia de un curso de preparación para emergencias asistido por ordenador para enfermeras de programas escolares.	N = 42 enfermeras de programas escolares	Teoría del aprendizaje cognitivo situado^(29)^.	Curso en línea sobre instrucción asistida por ordenador; evalúa la seguridad en la escena y las habilidades de estabilización.	15 módulos en línea de preparación para emergencias centrados en la toma de decisiones con retroalimentación inmediata y demostraciones de habilidades.	Los conocimientos y habilidades se evaluaron mediante un examen, una encuesta de confianza y un OMES, y los vídeos fueron evaluados por médicos ajenos al grupo.
3 Heidi Putman-Casdorph and Susan Pinto 2011, Estados Unidos^(30)^.	Diseño cuasi-experimental con evaluación previa y posterior a la intervención	Desarrollar y evaluar el aprendizaje a distancia para la formación continua en asma de las enfermeras escolares de Virginia Occidental.	N= 20 enfermeras de programas escolares.	Información básica sobre el asma según el programa National Asthma Education Prevention de los Institutos Nacionales de la Salud. Pautas para el cuidado del asma^(31)^.	Módulo piloto de educación sobre el asma: Impartido mediante tecnología de conferencia web (Wimba Live Classroom). Fisiopatología del asma, medicamentos y dispositivos de tratamiento.	Grupo 1: Recibió un módulo grabado de educación sobre el asma. Grupo 2: Recibió el módulo en directo con un instructor en línea. Las enfermeras obtuvieron unidades de formación continuada al finalizar.	Los conocimientos sobre el asma y la confianza en sí mismo se evaluaron mediante el SHPQ, que completaron los grupos de intervención antes y después del módulo, y el grupo de control por separado.
4 Cicutto et al., 2016, Estados Unidos^(32)^.	Un Delphi modificado y una evaluación del programa con intervención previa y posterior.	Desarrollar y aplicar un marco basado en competencias para un plan de estudios de formación continua para enfermeros escolares.	N=40 enfermeras de programas escolares.	Las competencias del National Asthma Educator Certification Board’s Certified Asthma Educator^(33)^ y el Healthy Learner Model^(34,35)^.	El modelo híbrido combinaba un programa en línea sobre el asma con un taller local, con 12 unidades de autoa-prendizaje en el sitio web de la Asthma and Allergy Foundation. Los participantes completaron la forma-ción a su propio ritmo.	Enfermeras de programas escolares completaron el curso en línea Manejo del asma y asistieron a talleres locales sobre cuidados del asma, con estudios de casos y evaluaciones prácticas de inhaladores.	Se evaluaron los conocimientos, la autoconfianza y las habilidades con el inhalador, y la autoeficacia se valoró de 0 a 4. El curso en línea incluía un cuestionario y evaluaba la satisfacción.
5 Lisa Blackmon-Jones, 2016, Estados Unidos^(36)^.	Evaluación del programa con intervención previa y posterior.	Evaluación del programa con intervención previa y posterior. Compartir los hallazgos de la implementación de un enfoque estandarizado y combinado para la orientación de Enfermeras de programas escolares.	N=20 enfermeras de programas escolares.	Según el marco teórico de Patricia Benner^(37)^.	La orientación mixta abarcó diabetes, asma, anafilaxia, cateterismos, alimentación por sonda, convulsiones, ventiladores, traqueostomía y salud mental.	El programa presenta estrategias como clases tradicionales y laboratorios de habilidades, con certificaciones de evaluación exigidas por el Estado que se completan al principio del año para un seguimiento eficaz.	Los participantes observaron mejoras en la formación y la tutoría. Para recolectar datos se realizaron autoevaluaciones, y una enfermera especialista evaluó la competencia tras la orientación.
6 Rhodes et al., 2019, Estados Unidos^(38)^.	Evaluación del programa con intervención previa y posterior.	Evaluar la eficacia de un módulo de aprendizaje electrónico rápido para el desarrollo profesional de las enfermeras escolares en el control escolar de la diabetes.	N=1127 enfermeras de programas escolares.	Las últimas directrices del Programa Nacional de Educación Diabética para el cuidado de la diabetes en la escuela se diseñaron utilizando el marco ICARE para el aprendizaje electrónico en la formación de enfermería^(39)^.	El módulo de aprendizaje electrónico rápido abordó la gestión de la diabetes en las escuelas, incluyendo actualizaciones sobre equipamiento, prácticas dietéticas, impactos psicológicos, gestión del tipo 2 y tres niveles de formación del personal.	El correo electrónico incluía un enlace, una carta de presenta-ción, un módulo de aprendizaje electrónico rápido con una prueba pre-post y un certificado. Un recordatorio animaba a completarlo en verano, y la fase Extend proporcionaba enlaces a recursos médicos fiables.	Los conocimientos sobre el control de la diabetes se evaluaron con preguntas de opción múltiple y verdadero-falso del módulo de aprendizaje electrónico, que incluía secciones de «cómo hacerlo» y comprobaciones de comprensión.
7 Shimasaki et al., 2019, Estados Unidos^(40)^.	Evaluación del programa con intervención previa y posterior.	Evaluar el impacto de la serie «Enfermeras de programas escolares que gestionan los cuidados de la diabetes» en la colaboración y la autoeficacia de las enfermeras escolares de Colorado.	N= 60 enfermeras de programas escolares.	El Framework for 21st Century School Nursing Practice™ centrado en el alumno destaca la enfermería centrada en el alumno, apoyada en principios profesionales clave^(41)^ y en directrices para el control intensivo de la diabetes ^(42)^.	El programa incluía sesiones de telesalud sobre atención diabética, con una serie ECHO de 4 semanas de dos cohortes, cada una con cuatro sesiones semanales de 1 hora.	Las sesiones apoyan el aprendizaje entre iguales mediante la presentación de casos y breves charlas didácticas, y están moderadas por un moderador, que permite hacer preguntas en directo o por chat.	Las sesiones promueven el aprendizaje entre iguales, con calificaciones de autoeficacia y satisfacción, comentarios abiertos sobre cambios en la práctica y obstáculos, y un cuestionario en línea a través de Qualtrics.
8 American Diabetes Association (ADA) 2021, Estados Unidos^(43)^.	Página web de formación.	Preparar y formar al personal escolar para que preste la atención necesaria a los alumnos con diabetes.		Guía actualizada en línea para ayudar al alumno con diabetes a tener éxito: guía para el personal escolar.	El sitio web ofrece recursos de autoaprendizaje para el personal escolar sobre el cuidado de la diabetes, que incluyen orientación, talleres y herramientas.	ADA School Diabetes Care Tasks es un programa de formación de 19 módulos dirigido a enfermeras de programas escolares para formar a personal no clínico en el cuidado de la diabetes.	Al final de cada módulo del programa Tareas para el cuidado de la diabetes en la escuela hay disponible un post-test: Lo que el personal clave debe saber.

Los estudios revisados abarcan desde 2008 hasta 2021, con la mayoría realizados después de 2016 (n = 5) y todos en los Estados Unidos de América (EE.UU.). La mayoría aplicó un enfoque cuantitativo (n = 6) para evaluar la eficacia de los programas de formación^(24,28,30,36,38,40)^ , mientras que uno utilizó un enfoque de métodos mixtos^(32)^. Al tratarse de un programa del sitio web de la American Diabetes Association, el artículo no especifica el tipo de estudio^(43)^.

Siete programas de formación abordaron enfermedades crónicas. Cuatro se centraron en la diabetes^(24,38,40,43)^, dos en el asma^(30,32)^, uno abarcó varias afecciones crónicas y otro, la preparación para emergencias^(28,36)^. Todos los estudios se centraron en enfermeras de salud escolar y se adhirieron a marcos teórico-metodológicos, incluyendo guías de práctica clínica^(24,30,32,38,40,43)^, referencias teóricas de enfermería y otras disciplinas^(24,28,32,36,40)^, y modelos educativos innovadores utilizando tecnología^(24,38)^.

Los objetivos generales de los estudios recolectados se centraron principalmente en la evaluación de la eficacia y en el desarrollo de formación apoyada en la tecnología^(24,28,30,32,38,40)^. Los objetivos específicos se centraban en el tratamiento de la diabetes^(24,38,40)^, el tratamiento del asma^(32)^ y otras enfermedades crónicas^(36)^, mientras que algunos estudios carecían de objetivos explícitos^(28,30,43)^.

La estructura del programa incluía temas como el control de la diabetes^(24,38,40,43)^, procedimientos de emergencia^(28)^, control del asma^(30,32)^ y procedimientos para afecciones crónicas^(36)^. Los programas se clasificaron en formatos de aprendizaje electrónico y semipresencial, y la mayoría de ellos se impartieron de forma asíncrona^(24,30,38,43)^, con sesiones síncronas ofrecidas a través de videoconferencia^(28,30,40)^. Todos ellos se organizaron en módulos temáticos de duración variable. Predominaron las actividades formativas breves, como un día de formación continua, sesiones de dos a diez horas y cuatro sesiones semanales de una hora cada una^(28,30,32,40)^. El aprendizaje tuvo lugar a lo largo de un periodo de ocho meses dentro de un único programa^(36)^. En un estudio no se especificaba el tiempo necesario para completar las actividades, pero se recomendaba completarlas durante el verano^(38)^. El contenido del sitio web de la ADA era accesible un número ilimitado de veces, lo que permitía a los alumnos establecer su propio ritmo de aprendizaje.

Las estrategias educativas se basaron en la tecnología, como los sistemas de gestión del aprendizaje^(24,30,32)^, los recursos de correo electrónico personalizados^(38)^ y las herramientas de colaboración en línea^(24)^. Se hizo hincapié en el aprendizaje basado en casos mediante sesiones tanto asíncronas como síncronas^(40)^. Se utilizaron escenarios audiovisuales y retroalimentación inmediata para mejorar las experiencias de aprendizaje^(28)^. El sitio web de la ADA ofrecía una serie de recursos, como talleres virtuales, estudios de casos y listas de comprobación de competencias para enfermeras de programas escolares^(43)^.

Los formatos de aprendizaje mixto combinaban módulos en línea con talleres presenciales basados en problemas^(32)^. También se utilizaron estrategias innovadoras, como laboratorios de habilidades y formación en documentación por ordenador^(36)^. Dos estudios controlaron el número de participantes para garantizar la calidad^(24,40)^.

La evaluación se centró en la autoeficacia, la confianza, la adquisición de conocimientos y el desarrollo de habilidades^(24,28,30,32,36,38,40)^. La calidad del programa se evaluó en tres estudios^(24,36,40)^, aplicándose a menudo evaluaciones sumativas. Un estudio destacó las evaluaciones formativas^(24)^. Se emplearon diversas herramientas de evaluación, como escalas de Likert, preguntas de verdadero-falso y escalas analógicas visuales. Las evaluaciones sumativas incluyeron escenarios simulados filmados y gestión de casos ^(28,36)^.

## DISCUSIÓN

Este estudio mostró los elementos esenciales de los programas de formación mediados por tecnología para profesionales de la salud escolar. Aquí se destacan los objetivos y las preguntas de investigación. Aunque nuestra investigación fue global y abarcó tres idiomas, todos los estudios identificados se realizaron en EE.UU. y se centraron en enfermeras escolares. Es probable que esto esté relacionado con el papel diferenciado que desempeñan estos profesionales en el contexto estadounidense. En EE.UU., las enfermeras de programas escolares son esenciales para promover la salud, el bienestar y el éxito educativo de los alumnos, ya que a menudo son los únicos profesionales sanitarios en los centros escolares^(3,13,44,45)^. La NASN, creada en 1968, refuerza este enfoque estructurado proporcionando directrices como el modelo^21st^ Century School Nursing Practice™ y abogando por la presencia a tiempo completo de enfermeras escolares^(41,46)^. Con aproximadamente 132 300 enfermeras escolares en los EE. UU.^(47)^, la inversión continua en su desarrollo profesional está respaldada por sistemas nacionales de evaluación, como la Encuesta Nacional de Salud Infantil^(48)^, que informan las políticas de salud escolar. Por el contrario, Europa muestra un importante vacío en la investigación sobre este tema, a pesar de las recomendaciones de la Red Europea de Escuelas Promotoras de Salud, que hace hincapié en la necesidad urgente de seguir investigando y de aplicar políticas de integración de la salud en las escuelas^(49)^. Además, en otras regiones del mundo, como América Latina, África y partes de Asia, tampoco se han identificado programas de formación estructurados en este campo de la salud escolar.

Esta Revisión de alcance incluyó estudios publicados en portugués, inglés y español, con la intención de dar una visión global de las prácticas mundiales. Sin embargo, a pesar de este enfoque inclusivo, todos los estudios incluidos se originaron en los Estados Unidos, lo que demuestra un sesgo geográfico significativo en la literatura. Esta limitación refleja la sobrerrepresentación de los contextos anglófonos y pone de relieve la falta de estudios de regiones como América Latina, que comparten similitudes lingüísticas pero están infrarrepresentadas en el corpus de investigación global.

 América Latina presenta una oportunidad única para la investigación debido a su gran diversidad étnica y cultural y a sus distintos determinantes sociales de la salud^(50)^. La investigación en esta región ha sido escasa, no sólo en el ámbito de la formación en salud escolar, sino también en otros ámbitos de la salud pública^(51)^. Para llenar estas brechas es necesario un esfuerzo consciente por incluir diversas voces y resultados de la investigación, promoviendo así una comprensión más completa de los retos y las soluciones de la salud mundial. Ampliar el ámbito geográfico de la investigación es crucial, no sólo para garantizar la inclusión, sino también para adaptar las intervenciones a las circunstancias culturales y contextuales.

Los datos revelaron un importante vacío en las estructuras pedagógicas de los programas de formación mediados por tecnología. Aunque los programas integran la tecnología para el aprendizaje a distancia, la mayoría no se ajustan a los modelos pedagógicos establecidos, convirtiendo a menudo las prácticas tradicionales en formatos virtuales^(52)^. Sólo dos programas incorporan modelos pedagógicos: el modelo ICARE - Introducción, Conexión, Aplicación, Reflexión y Extensión^(39)^ y el modelo de Difusión de Innovaciones de Rogers^(27)^, destacando la importancia de fundamentar los programas mediados por tecnología en principios pedagógicos sólidos. El uso de la tecnología con fines educativos es esencial y requiere un apoyo pedagógico en las estructuras, los actores y las estrategias de enseñanza^(53)^. En un marco de e-actividades, el diseño de actividades en línea con estrategias pedagógicas intencionadas es crucial para promover el compromiso y la participación activa, yendo más allá de la simple transferencia de prácticas tradicionales a formatos digitales^(54)^. Del mismo modo, para que la educación en línea sea eficaz es necesario estructurar los cursos basándose en teorías pedagógicas que faciliten interacciones significativas^(55)^, como el modelo de Comunidad de Investigación^(56)^, asegurándose de que los programas potenciados por la tecnología sean algo más que innovadores: son pedagógicamente sólidos. Los programas educativos en línea se consideran enfoques innovadores y complementarios^(57)^, y el aprendizaje combinado, en particular, tiene el potencial de aumentar la eficacia y la eficiencia de las actividades educativas^(58)^.

Estos programas estaban fuertemente influenciados por un enfoque práctico, incorporando pautas basadas en directrices recomendadas. También se observó que la influencia de los modelos teóricos de enfermería, como el marco centrado en el estudiante de la 21st Century School Nursing Practice™^(47)^ y el modelo de Benner^(37)^, puede ser crucial para estructurar la práctica enfermera y enriquecer la experiencia educativa^(59)^.

 La definición de CSHCN no se limita a la presencia de un diagnóstico clínico, sino que abarca las consecuencias que una condición puede generar en diversos ámbitos, lo que hace que el concepto sea más amplio e inclusivo^(60)^. Sin embargo, los programas identificados en esta revisión de investigación tienden a centrarse en condiciones de salud específicas. Los programas analizados tienden a priorizar afecciones destacadas en el perfil epidemiológico de los niños que requieren intervenciones urgentes, como la diabetes y el asma, probablemente debido al alto riesgo de resultados graves o mortales^(61, 62, 63)^.

Los programas de formación fueron fundamentales en todos los estudios recolectados, promoviendo intervenciones educativas que proporcionaron a las enfermeras los conocimientos, las habilidades y la confianza necesarios para prestar cuidados de calidad. Estos hallazgos confirman el impacto positivo de la formación continuada en el mantenimiento de unos cuidados de enfermería de alto nivel^(64)^. La formación continua es esencial para la gestión eficaz de las necesidades sanitarias especiales en el entorno escolar, y ésta debe adaptarse a las necesidades específicas de las enfermeras^(65)^.

Las estrategias docentes aplicadas en los estudios iban desde módulos de PowerPoint hasta foros de debate interactivos, promoviendo tanto el aprendizaje autónomo como el colaborativo. Se hace hincapié en la necesidad de entornos que faciliten la interacción entre enfermeras, así como en la flexibilidad que proporcionan las tecnologías digitales. Además, el e-learning ha surgido como una alternativa eficaz para la formación continuada en enfermería, demostrando resultados positivos en términos de satisfacción, flexibilidad y relevancia de los contenidos^(45,66,67)^.

Todos los estudios analizados destacaron la importancia de detallar cómo se produce la evaluación de los conocimientos, el desarrollo de habilidades, la autoeficacia y la confianza profesional de las enfermeras, integrando las prácticas de evaluación en la planificación de la formación. La formación basada en competencias pretende mejorar los resultados en salud de la población^(68)^. Los resultados de esta revisión indican que tales programas pueden reforzar la autoeficacia y la confianza de las enfermeras escolares^(69)^. La autoeficacia influye en la percepción de la capacidad de modificar conductas, lo cual es crucial para la adquisición de habilidades clínicas^(70)^. Aunque no se mencionó, el modelo de evaluación de Kirkpatrick se utiliza ampliamente para medir la eficacia de la formación a través de cuatro niveles^(71)^.

Los resultados muestran que las evaluaciones realizadas se centraron predominantemente en el componente sumativo, descuidando la evaluación formativa, que es crucial para monitorear el proceso de aprendizaje durante el diseño y desarrollo de la formación. Además, la mera incorporación de las tecnologías a la evaluación no garantiza una transformación eficaz de los métodos de evaluación^(72)^. Esto subraya la urgencia de realizar adaptaciones en la evaluación en respuesta a los cambios en los entornos educativos derivados de la migración a espacios digitales^(73)^. También es fundamental explorar nuevos paradigmas de evaluación y autoevaluación en modelos de formación mediados por tecnología, reconociendo y valorando la diversidad de métodos de evaluación digital. Para ello, se recomienda integrar tareas y actividades de evaluación que simulen situaciones de la vida real^(72)^. En este contexto, la implementación de la evaluación continua refuerza la estructura del modelo de evaluación, enfatizando su función formativa y promoviendo el desarrollo de los alumnos al situarlos en el centro del proceso educativo.

Esta revisión tiene sus limitaciones, como por ejemplo, la búsqueda incluye solamente de estudios en inglés, portugués o español. A pesar de estas limitaciones, se identificaron ciertas lagunas de conocimiento, proporcionando ideas para futuras investigaciones y sugiriendo un enfoque para el desarrollo de programas de formación mediados por tecnología centrados en las necesidades especiales de salud en un contexto escolar.

## CONCLUSIÓN

Esta Revisión de alcance reveló implicaciones metodológicas y prácticas significativas para el desarrollo de programas de formación dirigidos a equipos escolares, centrados en las necesidades especiales de salud. El uso de múltiples recursos digitales enriquece la formación, fomentando el aprendizaje tanto individual como colaborativo. De cara al futuro, es crucial abordar la falta de un marco pedagógico sólido para los entornos digitales, la necesidad de seguir investigando en los diversos ámbitos asociados a las necesidades especiales de salud, en particular los que implican el apoyo de equipos escolares interdisciplinares, y de una formación adaptada a los formatos digitales, que incorpore herramientas de evaluación formativa coherentes y adecuadas.
